# Association Between Soft Drink Consumption and Mortality in 10 European Countries

**DOI:** 10.1001/jamainternmed.2019.2478

**Published:** 2019-09-03

**Authors:** Amy Mullee, Dora Romaguera, Jonathan Pearson-Stuttard, Vivian Viallon, Magdalena Stepien, Heinz Freisling, Guy Fagherazzi, Francesca Romana Mancini, Marie-Christine Boutron-Ruault, Tilman Kühn, Rudolf Kaaks, Heiner Boeing, Krasimira Aleksandrova, Anne Tjønneland, Jytte Halkjær, Kim Overvad, Elisabete Weiderpass, Guri Skeie, Christine L. Parr, J. Ramón Quirós, Antonio Agudo, Maria-Jose Sánchez, Pilar Amiano, Lluís Cirera, Eva Ardanaz, Kay-Tee Khaw, Tammy Y. N. Tong, Julie A. Schmidt, Antonia Trichopoulou, Georgia Martimianaki, Anna Karakatsani, Domenico Palli, Claudia Agnoli, Rosario Tumino, Carlotta Sacerdote, Salvatore Panico, Bas Bueno-de-Mesquita, W. M. Monique Verschuren, Jolanda M. A. Boer, Roel Vermeulen, Stina Ramne, Emily Sonestedt, Bethany van Guelpen, Pernilla Lif Holgersson, Konstantinos K. Tsilidis, Alicia K. Heath, David Muller, Elio Riboli, Marc J. Gunter, Neil Murphy

**Affiliations:** 1 School of Public Health, Physiotherapy and Sports Science, University College Dublin, Dublin, Ireland; 2 School of Agriculture and Food Science, University College Dublin, Dublin, Ireland; 3 Section of Nutrition and Metabolism, International Agency for Research on Cancer, Lyon, France; 4 Instituto de Investigación Sanitaria Illes Balears (IdISBa), University Hospital of Son Espases, Palma de Mallorca, Spain; 5 CIBER Fisiopatología de la Obesidad y Nutrición (CIBEROBN), Madrid, Spain; 6 School of Public Health, MRC-PHE Centre for Environment and Health, Imperial College London, London, United Kingdom; 7 Department of Epidemiology and Biostatistics, School of Public Health, Imperial College London, London, United Kingdom; 8 CESP, Faculté de Médecine, Université Paris-Sud, Faculté de Médecine, UVSQ, INSERM, Université Paris-Saclay, Villejuif, France; 9 Gustave Roussy, F-94805, Villejuif, France; 10 German Cancer Research Center (DKFZ), Division of Cancer Epidemiology, Heidelberg, Germany; 11 Department of Epidemiology, German Institute of Human Nutrition, Potsdam-Rehbrücke, Germany; 12 Nutrition, Immunity and Metabolism Start-up Lab, Department of Epidemiology, Potsdam-Rehbrücke, Germany; 13 Danish Cancer Society Research Center, Copenhagen, Denmark; 14 Department of Public Health, Aarhus University, Aarhus, Denmark; 15 Office of the Director, International Agency for Research on Cancer, Lyon, France; 16 Department of Community Medicine, University of Tromsø, The Arctic University of Norway, Tromsø, Norway; 17 Department of Nursing and Health Promotion, Oslo Metropolitan University, Oslo, Norway; 18 Public Health Directorate, Asturias, Spain; 19 Unit of Nutrition and Cancer, Cancer Epidemiology Research Program, Catalan Institute of Oncology-IDIBELL, L’Hospitalet de Llobregat, Barcelona, Spain; 20 Escuela Andaluza de Salud Pública, Instituto de Investigación Biosanitaria Granada, Granada, Spain; 21 CIBER de Epidemiología y Salud Pública (CIBERESP), Madrid, Spain; 22 Public Health Division of Gipuzkoa, BioDonostia Research Institute, San Sebastian, Spain; 23 Department of Epidemiology, Murcia Regional Health Council, IMIB-Arrixaca, Murcia, Spain; 24 Navarra Public Health Institute, Pamplona, Spain; 25 IdiSNA, Navarra Institute for Health Research, Pamplona, Spain; 26 University of Cambridge School of Clinical Medicine, Clinical Gerontology Unit, Addenbrooke’s Hospital, Cambridge, United Kingdom; 27 Cancer Epidemiology Unit, Nuffield Department of Population Health, University of Oxford, Oxford, United Kingdom; 28 Hellenic Health Foundation, Athens, Greece; 29 WHO Collaborating Center for Nutrition and Health, Unit of Nutritional Epidemiology and Nutrition in Public Health, Department of Hygiene, Epidemiology and Medical Statistics, School of Medicine, National and Kapodistrian University of Athens, Athens, Greece; 30 Pulmonary Medicine Department, School of Medicine, National and Kapodistrian University of Athens, Attikon University Hospital, Haidari, Greece; 31 Cancer Risk Factors and Life-Style Epidemiology Unit, Institute for Cancer Research, Prevention and Clinical Network—ISPRO, Florence, Italy; 32 Epidemiology and Prevention Unit, Fondazione IRCCS Istituto Nazionale dei Tumori, Milan, Italy; 33 Cancer Registry and Histopathology Department, Civic—M. P. Arezzo Hospital, ASP Ragusa, Ragusa, Italy; 34 Unit of Cancer Epidemiology, Città della Salute e della Scienza University—Hospital and Center for Cancer Prevention (CPO), Turin, Italy; 35 Dipartimento di Medicina Clinica e Sperimentale, Federico II University, Naples, Italy; 36 Department for Determinants of Chronic Diseases (DCD), National Institute for Public Health and the Environment (RIVM), Bilthoven, the Netherlands; 37 Department of Gastroenterology and Hepatology, University Medical Centre, Utrecht, the Netherlands; 38 Department of Social and Preventive Medicine, Faculty of Medicine, University of Malaya, Pantai Valley, Kuala Lumpur, Malaysia; 39 Centre for Nutrition, Prevention and Health Services, National Institute for Public Health and the Environment (RIVM), Bilthoven, the Netherlands; 40 Environmental Epidemiology, Institute for Risk Assessment Sciences (IRAS), Utrecht University, Utrecht, the Netherlands; 41 Nutritional Epidemiology, Department of Clinical Sciences Malmö, Lund University, Malmö, Sweden; 42 Department of Radiation Sciences, Oncology, Umeå University, Umeå, Sweden; 43 Department of Hygiene and Epidemiology, University of Ioannina School of Medicine, Ioannina, Greece

## Abstract

**Importance:**

Soft drinks are frequently consumed, but whether this consumption is associated with mortality risk is unknown and has been understudied in European populations to date.

**Objective:**

To examine the association between total, sugar-sweetened, and artificially sweetened soft drink consumption and subsequent total and cause-specific mortality.

**Design, Setting, and Participants:**

This population-based cohort study involved participants (n = 451 743 of the full cohort) in the European Prospective Investigation into Cancer and Nutrition (EPIC), an ongoing, large multinational cohort of people from 10 European countries (Denmark, France, Germany, Greece, Italy, the Netherlands, Norway, Spain, Sweden, and the United Kingdom), with participants recruited between January 1, 1992, and December 31, 2000. Excluded participants were those who reported cancer, heart disease, stroke, or diabetes at baseline; those with implausible dietary intake data; and those with missing soft drink consumption or follow-up information. Data analyses were performed from February 1, 2018, to October 1, 2018.

**Exposure:**

Consumption of total, sugar-sweetened, and artificially sweetened soft drinks.

**Main Outcomes and Measures:**

Total mortality and cause-specific mortality. Hazard ratios (HRs) and 95% CIs were estimated using multivariable Cox proportional hazards regression models adjusted for other mortality risk factors.

**Results:**

In total, 521 330 individuals were enrolled. Of this total, 451 743 (86.7%) were included in the study, with a mean (SD) age of 50.8 (9.8) years and with 321 081 women (71.1%). During a mean (range) follow-up of 16.4 (11.1 in Greece to 19.2 in France) years, 41 693 deaths occurred. Higher all-cause mortality was found among participants who consumed 2 or more glasses per day (vs consumers of <1 glass per month) of total soft drinks (hazard ratio [HR], 1.17; 95% CI, 1.11-1.22; *P* < .001), sugar-sweetened soft drinks (HR, 1.08; 95% CI, 1.01-1.16; *P* = .004), and artificially sweetened soft drinks (HR, 1.26; 95% CI, 1.16-1.35; *P* < .001). Positive associations were also observed between artificially sweetened soft drinks and deaths from circulatory diseases (≥2 glasses per day vs <1 glass per month; HR, 1.52; 95% CI, 1.30-1.78; *P* < .001) and between sugar-sweetened soft drinks and deaths from digestive diseases (≥1 glass per day vs <1 glass per month; HR, 1.59; 95% CI, 1.24-2.05; *P* < .001).

**Conclusions and Relevance:**

This study found that consumption of total, sugar-sweetened, and artificially sweetened soft drinks was positively associated with all-cause deaths in this large European cohort; the results are supportive of public health campaigns aimed at limiting the consumption of soft drinks.

## Key Points

**Question:**

Is regular consumption of soft drinks associated with a greater risk of all-cause and cause-specific mortality?

**Findings:**

In this population-based cohort study of 451 743 individuals from 10 countries in Europe, greater consumption of total, sugar-sweetened, and artificially sweetened soft drinks was associated with a higher risk of all-cause mortality. Consumption of artificially sweetened soft drinks was positively associated with deaths from circulatory diseases, and sugar-sweetened soft drinks were associated with deaths from digestive diseases.

**Meaning:**

Results of this study appear to support ongoing public health measures to reduce the consumption of soft drinks.

## Teaser

This cohort study examines whether soft drink consumption is associated with all-cause and cause-specific mortality in a large cohort of European adults.

## Introduction

The frequent consumption of sugar-sweetened soft drinks increases energy intake, which can lead to weight gain and obesity.^1,2,3^ In 2010, the worldwide burden of adiposity-associated cardiovascular diseases, cancers, and type 2 diabetes associated with consumption of sugar-sweetened soft drinks was estimated to be 184 000 deaths.^4^ Reformulation of sugar-sweetened soft drinks, in which sugar is replaced with low- or no-calorie sweeteners, is being driven by consumer awareness and fiscal instruments, such as taxes.^5^ Artificially sweetened soft drinks have few or no calories; however, their long-term physiological and health implications are largely unknown.^6,7,8^

Whether regular consumption of soft drinks (total, sugar-sweetened, or artificially sweetened) is associated with greater all-cause and cause-specific mortality is uncertain, given that inconsistent findings were reported from previous prospective studies.^9,10^ Recently, a joint analysis of the Health Professionals Follow-up Study (HPFS) and Nurses’ Health Study (NHS) reported that a higher level of consumption of sugar-sweetened and artificially sweetened beverages was associated with greater all-cause mortality in the United States.^11^ Similarly, a positive association between artificially sweetened beverage consumption and all-cause mortality among US-based women was also reported by the Women’s Health Initiative Observational Study.^12^ Large-scale studies are required to examine the association between soft drink consumption and health outcomes, in particular research based in populations outside of the United States. To our knowledge, a large-scale European-based analysis of soft drink consumption and mortality outcomes has not been undertaken.

For cause-specific mortality, previous studies on soft drink consumption and cardiovascular disease mortality have found positive associations.^11,13^ However, whether these associations differed by type of circulatory diseases (eg, ischemic heart disease and cerebrovascular diseases) is uncertain. Similarly, scant data are available to date on mortality from other major chronic conditions such as cancers, digestive diseases, and neurodegenerative diseases.

We investigated the association of consuming total, sugar-sweetened, and artificially sweetened soft drinks with total and cause-specific mortality among participants in the European Prospective Investigation into Cancer and Nutrition (EPIC), an ongoing, large multinational cohort with more than 41 000 recorded deaths reported during the follow-up period.

## Methods

### Study Population

EPIC is a multicenter cohort of 521 330 participants who were recruited between January 1, 1992, and December 31, 2000, predominantly from the general populations of 10 European countries (Denmark, France, Germany, Greece, Italy, the Netherlands, Norway, Spain, Sweden, and the United Kingdom).^14,15^ Written informed consent was provided by all study participants. Ethical approval for this study was provided by the International Agency for Research on Cancer and the institutional review boards of the local participating EPIC centers.

For the current analysis, we excluded participants who at baseline reported cancer (n = 22 537), heart disease (n = 12 619), stroke (n = 3683), or diabetes (n = 12 461); participants in the highest or lowest 1% of the distribution of the ratio between energy intake to estimated energy requirement (ie, those with implausible dietary intake data; n = 8828); and participants with missing soft drink consumption or missing follow-up information (n = 9459). The final study cohort included 451 743 participants (130 662 [28.9%] men; 321 081 [71.1%] women).

### Assessment of Exposure

Dietary intake was assessed during the baseline enrollment visit (1992-2000) by country-specific instruments that were developed and validated within the various source populations in EPIC.^14,15^ Self-administered questionnaires were used in all centers, except in Greece, Spain, and Ragusa (Italy), where data were collected during personal interviews. In Malmö (Sweden), a combined semiquantitative food frequency questionnaire and 7-day dietary diary and diet interview was used. For soft drink consumption, participants recorded the number of glasses per month, week, or day; the structure of the questions varied somewhat by country and questionnaire. The dietary questionnaires for most countries collected information on the frequency of consumption (per glass) of “low calorie or diet fizzy soft drinks,” “fizzy soft drinks, eg cola, lemonade,” and “fruit squash or cordial.” Soft drink consumption (grams per day, which is roughly equivalent to the amount in milliliters; 1 glass was equal to approximately 250 mL) was calculated using typical glass sizes in each center.

Total soft drinks referred to a combination of soft drinks, carbonated and isotonic drinks, and diluted syrups. Total soft drink consumption was subdivided into sugar-sweetened and artificially sweetened soft drink consumption for all countries except Italy, Spain, and Sweden, where types of soft drinks were unmeasured. The reproducibility and validity of the dietary questionnaires were assessed in some countries,^14,15,16,17,18,19,20,21^ with correlations between repeated dietary questionnaires and with 24-hour dietary records ranging from 0.46 to 0.77 for soft or nonalcoholic drinks in the Netherlands, France, Germany, and Spain. Lifestyle questionnaires, administered at recruitment, were used as a source of information on educational attainment, smoking habits, alcohol intake, physical activity, reproductive and menstrual characteristics, and other variables.

### Ascertainment of Deaths

Data on vital status as well as the cause and date of death were collected by EPIC centers through record linkages with cancer registries, boards of health, and death indices in Denmark, Italy, the Netherlands, Norway, Spain, Sweden, and the United Kingdom or through active follow-up (inquiries by mail or telephone to municipal registries or regional health departments or to physicians or hospitals) in Germany, Greece, and France. For the current study, follow-up of participants from baseline (1992-2000) occurred from December 2009 to December 2013 for countries with record linkage. The end of follow-up was considered to be the last known contact with participants in France (June 2008), Germany (December 2009), and Greece (December 2012). Loss to follow-up was relatively low at 1.5%.

*International Statistical Classification of Diseases and Related Health Problems, Tenth Revision* (*ICD-10*) codes were used to classify the underlying cause of death. Deaths were grouped into common causes: cancer (*ICD-10* codes C00-D48), circulatory diseases (*ICD-10* codes I00-I99), and digestive diseases (*ICD-10* codes K00-K93). Additional specific causes were breast cancer (*ICD-10* code C50); colorectal cancer (*ICD-10* code C18-C20); prostate cancer (*ICD-10* code C61); cerebrovascular disease (*ICD-10* codes I60-I69); ischemic heart disease (*ICD-10* codes I20-I25); and the neurodegenerative diseases, Alzheimer (*ICD-10* code G30) and Parkinson (*ICD-10* code G20).

### Statistical Analysis

Hazard ratios (HRs) and 95% CIs for mortality were estimated using Cox proportional hazards regression models with age as the primary time metric. Time at study entry was age at recruitment, and exit time was age at death or the last date at which follow-up was considered complete in each EPIC center. Models were stratified by age at recruitment in 1-year categories, sex, and EPIC center. Soft drink consumption was categorized by the frequency of glasses consumed (<1 glass per month, 1 to 4 glasses per month, >1 to 6 glasses per week, 1 to <2 glasses per day, or ≥2 glasses per day, with 1 glass being equal to 250 mL). Linear trend tests across exposure groups were evaluated using the median category variables as continuous terms. Multivariable models were adjusted for alcohol consumption; smoking status, intensity, and duration; body mass index (BMI) (calculated as weight in kilograms divided by height in meters squared); physical activity; educational status; menopausal status; ever use of menopausal hormone therapy; and dietary intakes of total energy, red and processed meats, coffee, fruit and vegetable juices, and fruits and vegetables. Further adjustment for dietary fiber intake resulted in virtually unchanged risk estimates, so this variable was not included in the final multivariable models. Sugar-sweetened and artificially sweetened soft drinks were also mutually adjusted.

The association between soft drink consumption and mortality was also assessed across subgroups of smoking status, BMI, physical activity, and alcohol consumption. Tests for interaction were performed with the likelihood ratio test of models with and without interaction terms. Heterogeneity across countries was explored using a meta-analysis approach.^22^ We further investigated the shape of the association between soft drink consumption and all-cause mortality using restricted cubic splines with knots defined by the midpoints of aforementioned categories. The proportional hazard assumption was satisfied using Schoenfeld residuals^23^ analyses.

In sensitivity analyses, we excluded BMI from the multivariable models to assess the potential mediating role of adiposity for the association between soft drinks and mortality. To investigate reverse causality, analyses were conducted excluding deaths within the first 8 years of follow-up. We examined the associations of sole consumption of sugar-sweetened and artificially sweetened soft drinks with mortality. In addition, we examined the associations between soft drink consumption and mortality according to the death ascertainment method (linkage or active follow-up), with nonconsumers of soft drinks as the reference group, and with adjustment of the multivariable models for the World Cancer Research Fund dietary score^24^ (rather than individual dietary covariates). As a negative control analysis, we also examined the associations between soft drink consumption and deaths from external causes. All statistical tests were 2-sided, and *P* < .05 was considered statistically significant. Data analyses were performed from February 1, 2018, to October 1, 2018.

## Results

### Patients and Characteristics

Of the EPIC cohort of 521 330 participants, 451 743 (86.7%) were included in the study, among whom were 321 081 women (71.1%) and 130 662 men (28.9%) with a mean (SD) age of 50.8 (9.8) years. After a mean (range) follow-up of 16.4 (11.1 in Greece to 19.2 in France) years, 41 693 deaths (18 302 men and 23 391 women) were recorded. Of these deaths, 18 003 (43.2%) were from cancers, 9106 (21.8%) from circulatory diseases, and 1213 (2.9%) from digestive diseases. Compared with low consumers of soft drinks (<1 glass per month, high consumers (≥2 glasses per day) were younger (mean [IQR] age at recruitment, 52.2 [46.4-58.4] years vs 50.5 [38.4-56.6] years), more likely to be current smokers (46 154 [20.5%] vs 4706 [29.1%]), and more likely to be physically active (34 907 [15.5%] vs 4501 [27.8%]) (Table 1).

**Table 1.** Baseline Characteristics of Participants

Variable	Soft Drink Consumption, Median (IQR)					
	Total		Artificially Sweetened		Sugar-Sweetened	
	<1 Glass^a^ per mo	≥2 Glasses^a^ per d	<1 Glass^a^ per mo	≥2 Glasses^a^ per d	<1 Glass^a^ per mo	≥2 Glasses^a^ per d
All participants, No.	225 543	16 200	246 065	6292	195 505	7402
All-cause deaths, No.	21 032	1869	22 789	737	17 685	831
Age at recruitment, y	52.2 (46.4-58.4)	50.5 (38.4-56.6)	52.1 (46.1-58.4)	51.2 (40.9-56.6)	52.2 (46.5-58.2)	50.7 (34.7-56.9)
Women, No. (%)	172 480 (76.5)	9864 (60.9)	184 656 (75.0)	4556 (72.4)	155 705 (79.6)	4278 (57.8)
BMI	24.4 (22.1-27.3)	25.5 (22.8-28.6)	24.1 (21.8-26.9)	26.1 (23.4-30.0)	24.0 (21.8-26.8)	24.7 (22.2-27.7)
Higher education (including university), No. (%)	59 032 (26.2)	3211 (19.8)	69 713 (28.3)	1303 (20.7)	58 208 (29.8)	1555 (21.0)
Current smoker, No. (%)	46 154 (20.5)	4706 (29.1)	49 491 (20.1)	1623 (25.8)	37 203 (19.0)	2201 (29.7)
Physically active, No. (%)^b^	34 907 (15.5)	4501 (27.8)	43 357 (17.6)	1752 (27.8)	33 794 (17.3)	2237 (30.2)
Total energy intake, kcal per d	1969 (1609-2393)	2231 (1802-2737)	1982 (1630-2399)	1973 (1612-2431)	1923 (1578-2335)	2357 (1939-2858)
Consumption, g per d						
Red and processed meat	66.2 (38.4-99.5)	75.2 (34.3-117.3)	67.6 (38.6-101.7)	68.8 (26.3-109.6)	66.5 (37.2-100.7)	77.0 (28.7-122.8)
Fruits and vegetables	422.0 (274.4-606.2)	350.2 (218.1-535.2)	394.6 (251.2-587.5)	385.7 (244.0-579.1)	401.3 (257.7-588.6)	346.4 (215.3-518.0)
Alcohol	5.8 (0.9-16.6)	5.6 (1.1-14.1)	6.0 (1.3-15.8)	6.3 (1.4-15.1)	6.3 (1.4-16.2)	6.1 (1.3-14.3)
Coffee	227.1 (77.0-500.0)	476.9 (103.3-856.9)	314.3 (140.0-542.9)	500.0 (151.2-900.0)	337.5 (140.0-573.0)	476.9 (85.7-900.0)
Fruit and vegetable juices	14.3 (0.0-85.7)	17.1 (1.7-100.1)	35.6 (3.4-106.3)	16.8 (1.7-94.3)	28.6 (1.7-104.3)	18.2 (3.4-107.3)
Ever-use of contraceptive pill, No. (%)^c^	97 195 (56.4)	6781 (68.7)	111 871 (60.6)	3295 (72.3)	97 036 (62.3)	3021 (70.6)
Ever-use of menopausal hormone therapy, No. (%)^c^	45 378 (26.3)	2462 (25.0)	51 889 (28.1)	1317 (28.9)	47 168 (30.3)	992 (23.2)
Postmenopausal, No. (%)^c^	78 158 (45.3)	3641 (36.9)	82 945 (44.9)	1730 (38.0)	70 066 (45.0)	1561 (36.5)

Abbreviation: IQR, interquartile range.

^a^ 1 glass is equal to approximately 250 mL.

^b^ Defined as those with a sedentary job with more than 1 hour of recreational activity per day, a standing job with more than 30 minutes of recreational activity per day, a physical job with at least some recreational activity, or a heavy manual job.

^c^ Presented for women only.

### Soft Drink Consumption and Mortality

#### All-Cause Mortality

Higher all-cause mortality was found for participants who consumed 2 or more glasses per day (vs consumers of <1 glass per month) of total soft drinks (HR, 1.17; 95% CI, 1.11-1.22; *P* < .001), sugar-sweetened soft drinks (HR, 1.08; 95% CI, 1.01-1.16; *P* = .004), and artificially sweetened soft drinks (HR, 1.26; 95% CI, 1.16-1.35; *P* < .001) (Table 2). Similar associations were found for men and women (Table 2). Nonlinear J-shaped associations (nonlinear *P* < .001) were observed between all-cause mortality and total, sugar-sweetened, and artificially sweetened soft drinks, with higher risks observed at consumption levels of more than 125 mL per day for artificially sweetened soft drinks and more than 225 mL per day of sugar-sweetened soft drinks (eFigure in the Supplement). Among participants with a BMI lower than 25 (healthy weight), positive associations with all-cause mortality were found for total soft drinks (≥1 glass per day vs <1 glass per month; HR, 1.18; 95% CI, 1.11-1.25), sugar-sweetened soft drinks (HR, 1.11; 95% CI, 1.03-1.21), and artificially sweetened soft drinks (HR, 1.27; 95% CI, 1.12-1.43) (Figure).

**Table 2.** Associations Between Categories of Soft Drink Consumption and All-Cause Mortality

Variable	HR (95% CI)					*P* Value for Trend
	<1 Glass^a^ per mo	1 to 4 Glasses^a^ per mo	>1 to 6 Glasses^a^ per wk	1 to <2 Glasses^a^ per d	≥2 Glasses^a^ per d	
**Total Soft Drinks**						
Deaths, No.	21 032	5845	10 730	2217	1869	NA
Sexes combined						
Basic model^b^	1 [Reference]	0.95 (0.92-0.97)	0.98 (0.95-1.00)	1.16 (1.11-1.22)	1.26 (1.20-1.32)	<.001
Multivariable model^c^	1 [Reference]	0.97 (0.94-1.00)	0.98 (0.96-1.01)	1.10 (1.06-1.16)	1.17 (1.11-1.22)	<.001
Men						
Multivariable model^c^	1 [Reference]	0.96 (0.92-1.00)	0.99 (0.95-1.02)	1.09 (1.03-1.17)	1.16 (1.09-1.24)	<.001
Women						
Multivariable model^c^	1 [Reference]	0.97 (0.93-1.01)	0.98 (0.95-1.02)	1.12 (1.05-1.19)	1.16 (1.08-1.25)	<.001
**Artificially Sweetened Soft Drink** ^**d**^						
Deaths, No.	22 789	2679	2689	151	737	NA
Sexes combined						
Basic model^b^	1 [Reference]	0.92 (0.88-0.96)	1.01 (0.97-1.05)	1.09 (0.93-1.28)	1.35 (1.25-1.45)	<.001
Multivariable model^c^	1 [Reference]	0.93 (0.89-0.97)	1.01 (0.97-1.05)	0.99 (0.84-1.17)	1.26 (1.16-1.35)	<.001
Men						
Multivariable model^c^	1 [Reference]	0.94 (0.88-1.00)	1.06 (0.99-1.13)	1.12 (0.86-1.44)	1.26 (1.12-1.41)	<.001
Women						
Multivariable model^c^	1 [Reference]	0.93 (0.88-0.99)	0.97 (0.92-1.03)	0.92 (0.75-1.13)	1.24 (1.13-1.37)	<.001
**Sugar-Sweetened Soft Drink** ^**d**^						
Deaths, No.	17 685	4175	5420	934	831	NA
Sexes combined						
Basic model^b^	1 [Reference]	0.91 (0.88-0.95)	0.96 (0.93-1.00)	1.14 (1.07-1.23)	1.16 (1.08-1.25)	<.001
Multivariable model^c^	1 [Reference]	0.94 (0.91-0.98)	0.96 (0.93-1.00)	1.08 (1.01-1.16)	1.08 (1.01-1.16)	.004
Men						
Multivariable model^c^	1 [Reference]	0.96 (0.91-1.01)	0.96 (0.91-1.01)	1.03 (0.93-1.13)	1.09 (0.99-1.20)	.05
Women						
Multivariable model^c^	1 [Reference]	0.93 (0.89-0.98)	0.97 (0.93-1.01)	1.14 (1.04-1.25)	1.06 (0.95-1.18)	.04

Abbreviations: HR, hazard ratio; NA, not applicable.

^a^ One glass is equal to approximately 250 mL.

^b^ Basic Cox regression model adjusted for total energy intake (kcal per day) and stratified by age (1-year categories), EPIC (European Prospective Investigation into Cancer and Nutrition) center, and sex.

^c^ Multivariable Cox regression model adjusted for body mass index, calculated as weight in kilograms divided by height in meters squared (<22, 22 to <25, 25 to <30, 30 to <35, or ≥35); physical activity index (inactive, moderately inactive, moderately active, or active); educational status (none; primary school completed; technical or professional school; secondary school; longer education, including university; or not specified); alcohol consumption (nonconsumer, <5, 5 to <15, 15 to <30, or ≥30 g per day); smoking status and intensity (never; current: 1-15 cigarettes per day; current: 16-25 cigarettes per day; current: ≥16 cigarettes per day; former: quit ≤10 y; former: quit 11-20 y; former: quit ≥20 y; current: pipe, cigar, occasional; current or former: missing; or unknown); smoking duration (<10, 10 to <20, 20 to <30, 30 to <40, ≥40 y, or smoking duration unknown); ever use of contraceptive pill (yes, no, or unknown); menopausal status (premenopausal, postmenopausal, perimenopausal or unknown menopausal status, or surgical postmenopausal); ever use of menopausal hormone therapy (yes, no, or unknown); and intakes of total energy (kcal per day), red and processed meat (g per day), fruits and vegetables (g per day), coffee (g per day), and fruit and vegetable juice (g per day) (all continuous); and stratified by age (1-year categories), EPIC center, and sex.

^d^ Sugar-sweetened and artificially sweetened soft drinks were mutually adjusted. Italy, Spain, and Sweden were excluded from these analyses because information on type of soft drink consumption was not collected.

**Figure.**
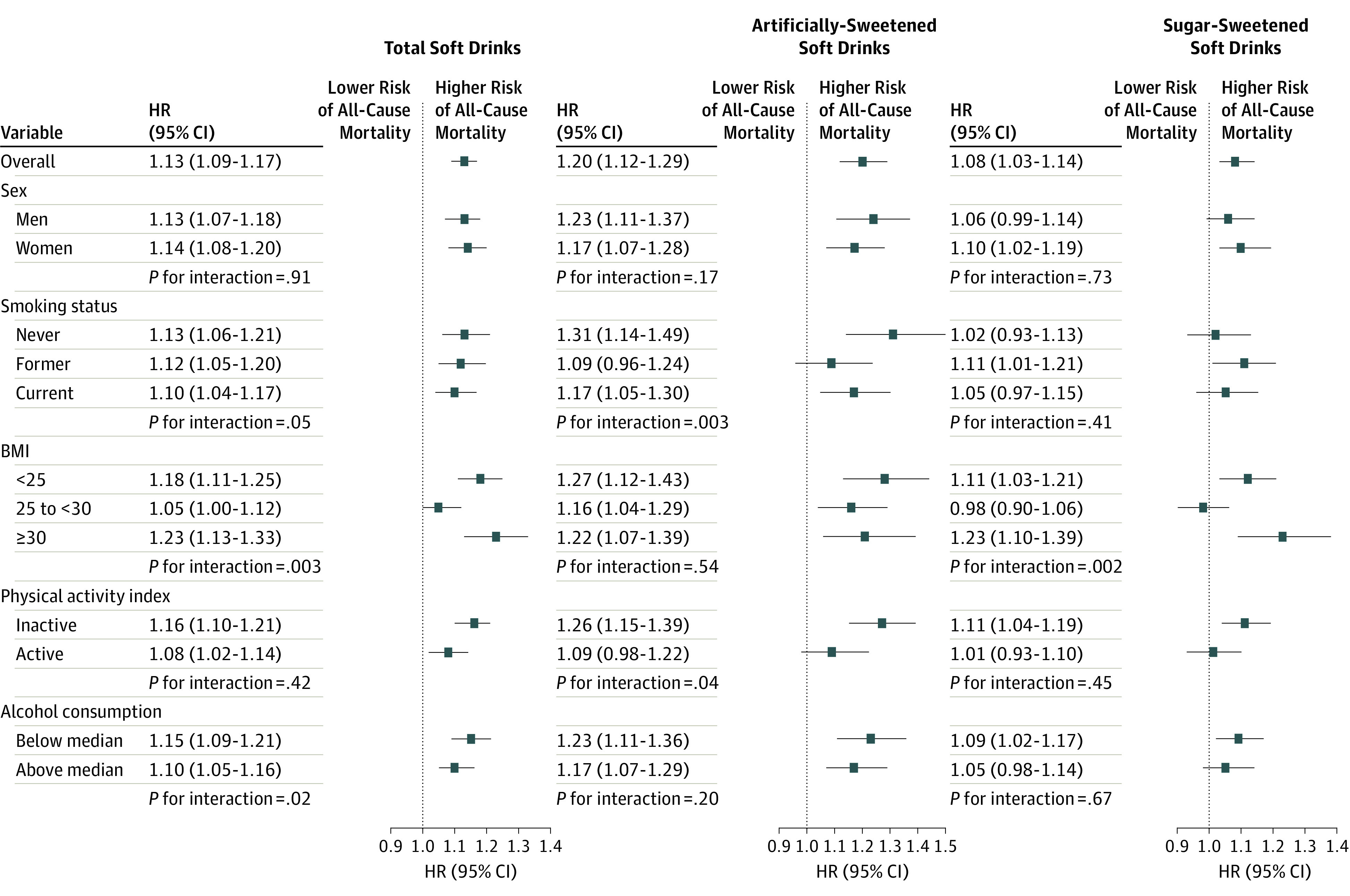
Subgroup Analyses of the Association Between Soft Drink Consumption and All-Cause Mortality The hazard ratios (HRs) are for comparing participants who drank ≥1 glass per day with those who had <1 glass per month. Multivariable Cox regression model adjusted for body mass index (BMI), calculated as weight in kilograms divided by height in meters squared (<22, 22 to <25, 25 to <30, 30 to <35, or ≥35); physical activity index (inactive, moderately inactive, moderately active, or active); educational status (none; primary school completed; technical or professional school; secondary school; longer education, including university; or not specified); alcohol consumption (nonconsumer, <5, 5 to <15, 15 to <30, or ≥30 g per day); smoking status and intensity (never; current: 1-15 cigarettes per day; current: 16-25 cigarettes per day; current: ≥16 cigarettes per day; former: quit ≤10 y; former: quit 11-20 y; former: quit ≥20 y; current: pipe, cigar, occasional; current or former: missing; or unknown); smoking duration (<10, 10 to <20, 20 to <30, 30 to <40, ≥40 y, or smoking duration unknown); ever use of contraceptive pill (yes, no, or unknown); menopausal status (premenopausal, postmenopausal, perimenopausal or unknown menopausal status, or surgical postmenopausal); ever use of menopausal hormone therapy (yes, no, or unknown); and intakes of total energy (kcal per day), red and processed meat (g per day), fruits and vegetables (g per day), coffee (g per day), and fruit and vegetable juice (g per day) (all continuous); and stratified by age (1-year categories), EPIC center, and sex. Sugar-sweetened and artificially sweetened soft drinks were mutually adjusted. Italy, Spain, and Sweden were excluded from the sugar-sweetened and artificially sweetened soft drinks analyses because information on type of soft drink consumption was not collected. Median alcohol consumption was 5.4 g per day. EPIC indicates European Prospective Investigation into Cancer and Nutrition.

The soft drink consumption and all-cause mortality associations were generally consistent across subgroups of other mortality risk factors. For sugar-sweetened soft drinks, a positive association was found among participants with a BMI of 30 or higher (obese) but not among those with a BMI between 25 and under 30 (overweight) (HR, 1.23; 95% CI, 1.10-1.39 vs HR, 0.98; 95% CI, 0.90-1.06; *P* = .002; Figure). Results remained similar when country-specific estimates were pooled in a random-effects meta-analysis (eTable 1 in the Supplement). No heterogeneity across countries was found for artificially sweetened soft drinks and all-cause mortality (*I^2^* = 0%; *P* = .86); however, heterogeneity was detected for sugar-sweetened soft drinks (*I^2^* = 63%; *P* = .01), although positive associations were found for all but 1 country.

### Soft Drink Consumption and Cause-Specific Mortality

#### Circulatory Diseases

Higher circulatory diseases mortality risk was found for participants consuming 2 or more glasses per day (vs consumers of <1 glass per month) of total soft drinks (HR, 1.27; 95% CI, 1.14-1.40; *P* < .001) and artificially sweetened soft drinks (HR, 1.52; 95% CI, 1.30-1.78; *P* < .001) but not sugar-sweetened soft drinks (HR, 1.11; 95% CI, 0.95-1.30; *P* = .16) (Table 3). Similar results were found for men and women. Positive associations for ischemic heart disease mortality risk were found for total soft drinks (≥1 glass per day vs <1 glass per month; HR, 1.19; 95% CI, 1.06-1.33; *P* = .001) and artificially sweetened soft drinks (HR, 1.41; 95% CI, 1.11-1.79; *P* = .003) (Table 4), with no association for sugar-sweetened soft drinks. Total soft drinks were positively associated with cerebrovascular disease mortality risk (HR, 1.30; 95% CI, 1.12-1.50; *P* < .001), with positive statistically nonsignificant associations found for sugar-sweetened and artificially sweetened soft drinks (Table 4).

**Table 3.** Multivariable Associations Between Categories of Soft Drink Consumption and Mortality From Cancers, Circulatory Diseases, or Digestive Diseases^a^

Variable	HR (95% CI)					*P* Value for Trend
	<1 Glass^b^ per mo	1 to 4 Glasses^b^ per mo	>1 to 6 Glasses^b^ per wk	1 to <2 Glasses^b^ per d	≥2 Glasses^b^ per d	
**Cancers, *ICD-10* Codes C00-D48**						
Total soft drinks						
Deaths, No.	9029	2610	4787	845	732	NA
Sexes combined	1 [Reference]	0.99 (0.95-1.04)	1.00 (0.96-1.04)	1.02 (0.95-1.10)	1.02 (0.95-1.11)	.45
Men	1 [Reference]	1.01 (0.94-1.08)	1.03 (0.97-1.09)	1.01 (0.91-1.13)	1.05 (0.94-1.17)	.39
Women	1 [Reference]	0.99 (0.93-1.05)	0.98 (0.93-1.03)	1.03 (0.93-1.14)	1.00 (0.89-1.11)	.83
Artificially sweetened soft drinks^c^						
Deaths, No.	9359	1246	1251	72	303	
Sexes combined	1 [Reference]	0.96 (0.90-1.02)	1.00 (0.94-1.06)	0.92 (0.73-1.16)	1.10 (0.97-1.23)	.23
Men	1 [Reference]	0.99 (0.89-1.09)	1.10 (0.99-1.22)	1.13 (0.76-1.66)	1.14 (0.95-1.37)	.06
Women	1 [Reference]	0.95 (0.87-1.02)	0.95 (0.88-1.02)	0.83 (0.62-1.11)	1.06 (0.91-1.24)	.98
Sugar-sweetened soft drinks^c^						
Deaths, No.	7385	1797	2405	323	321	NA
Sexes combined	1 [Reference]	0.95 (0.90-1.01)	0.97 (0.92-1.02)	0.97 (0.86-1.09)	0.95 (0.84-1.06)	.33
Men	1 [Reference]	1.01 (0.93-1.09)	0.99 (0.92-1.08)	0.98 (0.83-1.17)	0.97 (0.83-1.14)	.69
Women	1 [Reference]	0.92 (0.85-0.98)	0.95 (0.89-1.01)	0.97 (0.83-1.14)	0.92 (0.78-1.09)	.32
**Circulatory Diseases, *ICD-10* Codes I00-I99**						
Total soft drinks						
Deaths, No.	4294	1272	2513	592	435	NA
Sexes combined	1 [Reference]	0.96 (0.90-1.02)	0.98 (0.93-1.03)	1.19 (1.09-1.31)	1.27 (1.14-1.40)	<.001
Men	1 [Reference]	0.96 (0.88-1.05)	0.95 (0.89-1.03)	1.18 (1.05-1.33)	1.23 (1.08-1.41)	<.001
Women	1 [Reference]	0.96 (0.87-1.05)	1.01 (0.94-1.10)	1.21 (1.05-1.38)	1.31 (1.11-1.54)	<.001
Artificially sweetened soft drinks^c^						
Deaths, No.	4614	531	525	27	170	NA
Sexes combined	1 [Reference]	0.91 (0.83-1.00)	1.01 (0.92-1.11)	1.02 (0.70-1.50)	1.52 (1.30-1.78)	<.001
Men	1 [Reference]	0.81 (0.71-0.94)	1.00 (0.87-1.15)	0.91 (0.51-1.61)	1.53 (1.23-1.91)	<.001
Women	1 [Reference]	1.00 (0.88-1.13)	1.01 (0.89-1.15)	1.13 (0.67-1.88)	1.50 (1.19-1.88)	.001
Sugar-sweetened soft drinks^c^						
Deaths, No.	3311	955	1206	220	175	NA
Sexes combined	1 [Reference]	0.97 (0.90-1.05)	0.96 (0.90-1.04)	1.06 (0.92-1.22)	1.11 (0.95-1.30)	.16
Men	1 [Reference]	0.99 (0.89-1.10)	0.94 (0.85-1.04)	0.98 (0.80-1.20)	1.11 (0.91-1.35)	.43
Women	1 [Reference]	0.97 (0.87-1.07)	0.99 (0.89-1.09)	1.15 (0.94-1.40)	1.11 (0.86-1.43)	.20
**Digestive Diseases, *ICD-10* Codes K00-K93** ^d^						
Total soft drinks						
Deaths, No.	567	171	319	156	NA	NA
Sexes combined	1 [Reference]	1.07 (0.89-1.28)	1.16 (1.00-1.34)	1.50 (1.24-1.81)	NA	<.001
Men	1 [Reference]	0.96 (0.74-1.25)	1.24 (1.01-1.52)	1.52 (1.17-1.96)	NA	.001
Women	1 [Reference]	1.17 (0.92-1.49)	1.07 (0.86-1.33)	1.45 (1.10-1.93)	NA	.02
Artificially sweetened soft drinks^c^						
Deaths, No.	662	88	91	24	NA	NA
Sexes combined	1 [Reference]	1.00 (0.79-1.27)	1.19 (0.95-1.50)	0.99 (0.65-1.50)	NA	.78
Men	1 [Reference]	1.13 (0.80-1.59)	1.23 (0.88-1.73)	1.04 (0.58-1.87)	NA	.74
Women	1 [Reference]	0.92 (0.67-1.27)	1.14 (0.83-1.55)	0.91 (0.51-1.64)	NA	.93
Sugar-sweetened soft drinks^c^						
Deaths, No.	494	133	158	80	NA	NA
Sexes combined	1 [Reference]	1.05 (0.86-1.28)	1.07 (0.88-1.29)	1.59 (1.24-2.05)	NA	<.001
Men	1 [Reference]	0.94 (0.70-1.27)	1.09 (0.83-1.43)	1.51 (1.06-2.14)	NA	.02
Women	1 [Reference]	1.15 (0.88-1.50)	1.04 (0.79-1.37)	1.67 (1.16-2.41)	NA	.01

Abbreviations: HR, hazard ratio; *ICD-10*, *International Statistical Classification of Diseases and Related Health Problems, Tenth Revision*; NA, not applicable.

^a^ Multivariable Cox regression model adjusted for body mass index, calculated as weight in kilograms divided by height in meters squared (<22, 22 to <25, 25 to <30, 30 to <35, or ≥35); physical activity index (inactive, moderately inactive, moderately active, or active); educational status (none; primary school completed; technical or professional school; secondary school; longer education, including university; or not specified); alcohol consumption (nonconsumer, <5, 5 to <15, 15 to <30, or ≥30 g per day); smoking status and intensity (never; current: 1-15 cigarettes per day; current: 16-25 cigarettes per day; current: ≥16 cigarettes per day; former: quit ≤10 y; former: quit 11-20 y; former: quit ≥20 y; current: pipe, cigar, occasional; current or former: missing; or unknown); smoking duration (<10, 10 to <20, 20 to <30, 30 to <40, ≥40 y, or smoking duration unknown); ever use of contraceptive pill (yes, no, or unknown); menopausal status (premenopausal, postmenopausal, perimenopausal or unknown menopausal status, or surgical postmenopausal); ever use of menopausal hormone therapy (yes, no, or unknown); and intakes of total energy (kcal per day), red and processed meat (g per day), fruits and vegetables (g per day), coffee (g per day), and fruit and vegetable juice (g per day) (all continuous); and stratified by age (1-year categories), EPIC (European Prospective Investigation into Cancer and Nutrition) center, and sex.

^b^ 1 glass is equal to approximately 250 mL.

^c^ Sugar-sweetened and artificially sweetened soft drinks were mutually adjusted. Italy, Spain, and Sweden were excluded from these analyses because information on type of soft drink consumption was not collected.

^d^ Top 2 categories were merged as ≥1 glasses per day because of few cases.

**Table 4.** Multivariable Associations Between Categories of Soft Drink Consumption and Mortality From Cause-Specific Cancer, Circulatory Disease, or Neurodegenerative Disease^a^

Variable	HR (95% CI)				*P* Value for Trend
	<1 Glass^b^ per mo	1 to 4 Glasses^b^ per mo	>1 to 6 Glasses^b^ per wk	≥1 Glass^b^ per d	
**Colorectal Cancer, *ICD-10* Codes C18-C20 (No. of Deaths = 2095)** ^c^					
Total soft drinks	1 [Reference]	1.00 (0.87-1.14)	1.05 (0.94-1.18)	1.25 (1.07-1.47)	.004
Artificially sweetened soft drinks^d^	1 [Reference]	1.08 (0.91-1.28)	1.02 (0.85-1.22)	1.22 (0.91-1.64)	.21
Sugar-sweetened soft drinks^d^	1 [Reference]	0.95 (0.81-1.11)	1.00 (0.86-1.15)	1.10 (0.86-1.40)	.41
**Breast Cancer, *ICD-10* Code C50 (No. of Deaths = 1402)** ^c^					
Total soft drinks	1 [Reference]	0.95 (0.80-1.12)	0.98 (0.85-1.13)	1.13 (0.92-1.39)	.20
Artificially sweetened soft drinks^d^	1 [Reference]	0.79 (0.63-0.98)	0.90 (0.74-1.10)	0.85 (0.59-1.22)	.38
Sugar-sweetened soft drinks^d^	1 [Reference]	0.87 (0.71-1.06)	1.07 (0.90-1.27)	1.21 (0.91-1.62)	.10
**Prostate Cancer, *ICD-10* Code C61 (No. of Deaths = 907)** ^c^					
Total soft drinks	1 [Reference]	1.03 (0.85-1.25)	1.03 (0.88-1.22)	0.97 (0.77-1.24)	.80
Artificially sweetened soft drinks^d^	1 [Reference]	1.23 (0.95-1.60)	1.36 (1.05-1.78)	1.05 (0.64-1.75)	.53
Sugar-sweetened soft drinks^d^	1 [Reference]	1.14 (0.91-1.42)	1.05 (0.84-1.32)	1.08 (0.77-1.51)	.76
**Cerebrovascular Diseases, *ICD-10* Codes I60-I69 (No. of Deaths = 2380)** ^c^					
Total soft drinks	1 [Reference]	1.00 (0.88-1.13)	0.97 (0.87-1.08)	1.30 (1.12-1.50)	<.001
Artificially sweetened soft drinks^d^	1 [Reference]	0.85 (0.71-1.03)	1.06 (0.89-1.27)	1.24 (0.91-1.70)	.12
Sugar-sweetened soft drinks^d^	1 [Reference]	0.97 (0.84-1.12)	0.99 (0.87-1.14)	1.19 (0.97-1.47)	.10
**Ischemic Heart Disease, *ICD-10* Codes I20-I25 (No. of Deaths = 3536)** ^c^					
Total soft drinks	1 [Reference]	0.94 (0.84-1.04)	0.99 (0.91-1.07)	1.19 (1.06-1.33)	.001
Artificially sweetened soft drinks^d^	1 [Reference]	0.89 (0.76-1.04)	1.06 (0.91-1.23)	1.41 (1.11-1.79)	.003
Sugar-sweetened soft drinks^d^	1 [Reference]	1.03 (0.91-1.16)	0.95 (0.85-1.07)	1.04 (0.87-1.23)	.84
**Parkinson Disease, *ICD-10* Code G20 (No. of Deaths = 254)** ^c^					
Total soft drinks	1 [Reference]	0.90 (0.60-1.33)	0.81 (0.58-1.14)	1.59 (1.07-2.36)	.02
Artificially sweetened soft drinks^d^	1 [Reference]	0.84 (0.48-1.49)	1.23 (0.73-2.06)	1.50 (0.64-3.48)	.27
Sugar-sweetened soft drinks^d^	1 [Reference]	0.91 (0.59-1.43)	0.90 (0.58-1.39)	1.39 (0.79-2.43)	.25
**Alzheimer Disease, *ICD-10* Code G30 (No. of Deaths = 453)** ^c^					
Total soft drinks	1 [Reference]	1.23 (0.93-1.62)	1.13 (0.89-1.44)	0.82 (0.53-1.26)	.33
Artificially sweetened soft drinks^d^	1 [Reference]	0.94 (0.57-1.55)	1.20 (0.72-1.98)	0.57 (0.14-2.33)	.59
Sugar-sweetened soft drinks^d^	1 [Reference]	1.11 (0.75-1.65)	1.35 (0.94-1.96)	0.90 (0.44-1.81)	.99

Abbreviations: HR, hazard ratio; *ICD-10*, *International Statistical Classification of Diseases and Related Health Problems, Tenth Revision*.

^a^ Multivariable Cox regression model adjusted for body mass index, calculated as weight in kilograms divided by height in meters squared (<22, 22 to <25, 25 to <30, 30 to <35, or ≥35); physical activity index (inactive, moderately inactive, moderately active, or active); educational status (none; primary school completed; technical or professional school; secondary school; longer education, including university; or not specified); alcohol consumption (nonconsumer, <5, 5 to <15, 15 to <30, or ≥30 g per day); smoking status and intensity (never; current: 1-15 cigarettes per day; current: 16-25 cigarettes per day; current: ≥16 cigarettes per day; former: quit ≤10 y; former: quit 11-20 y; former: quit ≥20 y; current: pipe, cigar, occasional; current or former: missing; or unknown); smoking duration (<10, 10 to <20, 20 to <30, 30 to <40, ≥40 y, or smoking duration unknown); ever use of contraceptive pill (yes, no, or unknown); menopausal status (premenopausal, postmenopausal, perimenopausal or unknown menopausal status, or surgical postmenopausal); ever use of menopausal hormone therapy (yes, no, or unknown); and intakes of total energy (kcal per day), red and processed meat (g per day), fruits and vegetables (g per day), coffee (g per day), and fruit and vegetable juice (g per day) (all continuous); and stratified by age (1-year categories), EPIC (European Prospective Investigation into Cancer and Nutrition) center, and sex.

^b^ One glass is equal to approximately 250 mL.

^c^ Number of deaths based on total soft drink consumption models.

^d^ Sugar-sweetened and artificially sweetened soft drinks were mutually adjusted. Italy, Spain, and Sweden were excluded from these analyses because information on type of soft drink consumption was not collected.

#### Cancer

Total, sugar-sweetened, and artificially sweetened soft drink consumption was not associated with risk of deaths from overall cancer (Table 3), breast cancer, or prostate cancer (Table 4). Total soft drink consumption was positively associated with colorectal cancer deaths (≥1 glass per day vs <1 glass per month; HR, 1.25; 95% CI, 1.07-1.47; *P* = .004) (Table 4), with statistically nonsignificant associations found for sugar-sweetened and artificially sweetened soft drinks.

#### Digestive Diseases

Higher level of consumption of total soft drinks and sugar-sweetened soft drinks (≥1 glass per day vs <1 glass per month; HR, 1.59; 95% CI, 1.24-2.05; *P* < .001), but not artificially sweetened soft drinks, was associated with digestive disease mortality. Similar associations were found for men and women (Table 3).

#### Neurodegenerative Diseases

Total soft drink consumption was positively associated with risk of Parkinson disease mortality (≥1 glass per day vs <1 glass per month; HR, 1.59; 95% CI, 1.07-2.36; *P* = .02), with similar magnitude nonsignificant associations found for artificially sweetened and sugar-sweetened soft drinks (Table 4). Soft drinks were not associated with Alzheimer disease mortality.

### Sensitivity Analyses

Sugar-sweetened and artificially sweetened soft drink consumption was positively associated with circulatory disease and digestive disease mortality among participants with a healthy weight (eTable 2 in the Supplement). Similar positive associations between soft drink consumption and mortality outcomes were found when the multivariable models excluded BMI adjustment (eTable 3 in the Supplement), deaths that occurred during the first 8 years of follow-up were excluded (eTable 4 in the Supplement), nonconsumers of soft drinks were set as the reference group (eTable 5 in the Supplement), and multivariable models were adjusted for the World Cancer Research Fund dietary score rather than individual dietary covariates (eTable 6 in the Supplement). A similar pattern of results for all mortality end points was found for sole consumers of artificially sweetened and sugar-sweetened soft drinks (eTable 7 in the Supplement). The positive sugar-sweetened association was stronger for countries with active follow-up, compared with those with linkage follow-up (eTable 8 in the Supplement). No associations were found between soft drink consumption and deaths by external causes (eTable 9 in the Supplement).

## Discussion

In this large multinational European study, higher level of consumption of total, sugar-sweetened, and artificially sweetened soft drinks was associated with increased risk of death from all causes. The positive association between soft drink consumption and mortality was evident for both men and women. Only artificially sweetened, and not sugar-sweetened, soft drinks were associated with deaths from circulatory diseases, whereas for digestive disease deaths, only sugar-sweetened soft drinks were associated with higher risk.

The high level of consumption of sugar-sweetened and artificially sweetened soft drinks has previously been linked to elevated risks of obesity, type 2 diabetes, and cardiovascular disease.^1,25,26,27^ We found that sugar-sweetened soft drinks were positively associated with all-cause mortality, a result consistent with findings from an HPFS/NHS analysis^11^ but inconsistent with findings from smaller Singapore- and US-based studies.^10,28^ We also found positive associations between consumption of artificially sweetened soft drinks and all-cause mortality, a finding consistent with results of the HPFS/NHS and Women’s Health Initiative studies.^11,12^ Overall, to our knowledge, this current study was the largest to date to investigate the associations between soft drink consumption and mortality outcomes as well as the first comprehensive European-based analysis.

We found nonlinear J-shaped associations for soft drink consumption and all-cause mortality, with higher risks observed at consumption levels of more than 125 mL per day (half a glass) of artificially sweetened soft drinks and more than 225 mL per day (approximately 1 glass) of sugar-sweetened soft drinks, and the lowest risks found from drinking 50 mL per day. The reduced risk found at low consumption levels may be the result of reverse causality (analogous to what has been observed for alcohol consumption and all-cause mortality^29,30,31^), driven by participants with disease symptoms reporting nonconsumption of soft drinks.

The role of BMI in the soft drink consumption and mortality outcomes association is complex, with adiposity likely a mediating and confounding factor that varies by cause of death. In our analyses, results were unchanged between the multivariable models with or without BMI adjustment, suggesting that the observed associations may be independent of adiposity. In support of this hypothesis, positive associations were found between total, sugar-sweetened, and artificially sweetened soft drinks with deaths from all causes, circulatory diseases, and digestive diseases among participants with healthy weight. These results may suggest that soft drinks alter mortality risk independently of adiposity, possibly because of the high glycemic index of sugar-sweetened soft drinks,^32^ which elevates blood glucose levels and may in turn lead to insulin resistance and inflammation.^33,34^

For sugar-sweetened soft drinks, we found a positive association with all-cause mortality for participants who were obese but no association for participants who were overweight. The lack of association among overweight participants is inconsistent with the result of an analysis of the HPFS/NHS study, which found a positive association with all-cause mortality among overweight adults.^11^ It is unclear why we observed a positive association for sugar-sweetened soft drinks among obese but not among overweight participants. We cannot exclude the role of chance in these subgroup analyses, and additional large-scale prospective studies are required to examine these associations further.

In cause-specific analyses, we found positive associations between artificially sweetened soft drink consumption and deaths from circulatory diseases; these results are consistent with those in the HPFS/NHS and Women’s Health Initiative analyses.^11,13^ These results were largely based on the positive association between ischemic heart disease deaths and consumption of artificially sweetened soft drinks. Reverse causality is a possible explanation for this positive association, with unhealthy individuals at the study baseline (eg, those who were overweight or obese; those with prediabetes) switching from drinking sugar-sweetened to artificially sweetened soft drinks to control their body weight. However, this association persisted when deaths recorded in the first 8 years of follow-up were excluded. In addition, positive associations between artificially sweetened soft drinks and all-cause and circulatory diseases mortality were found among participants with healthy weight. Possible biological mechanisms that may explain the positive associations between artificially sweetened soft drinks and mortality outcomes are unclear. Limited experimental evidence suggests that artificial sweeteners may induce glucose intolerance,^35^ but further studies are needed into the possible adverse effects of the long-term consumption of artificial sweeteners commonly used in soft drinks, such as aspartame and acesulfame potassium.^36^

A higher level of soft drink consumption was associated with greater risk of death from digestive diseases, with a positive association only found for sugar-sweetened soft drinks. Hyperglycemia resulting from consumption of sugar-sweetened soft drinks may alter gut-barrier function and increase the risk of enteric infection.^37^ Furthermore, fructose, a sugar commonly used in soft drinks, promotes liver lipogenesis, which can lead to nonalcoholic fatty liver disease and lower insulin sensitivity.^38,39,40^

We observed no association between soft drink consumption and overall cancer mortality. This result is consistent with findings in most previous studies, which found little evidence of a direct association between soft drink consumption and cancer risk,^2^ but it is inconsistent with that in the HPFS/NHS analysis, which reported a positive association between sugar-sweetened beverages and cancer mortality.^11^ We did observe a positive association between total soft drink consumption and risk of colorectal cancer mortality, but our analyses could not ascertain whether sugar-sweetened or artificially sweetened soft drinks were factors in this association. In the HPFS/NHS analysis, a borderline positive association was observed between sugar-sweetened beverages and colon cancer mortality.^11^ Further studies into soft drinks and cancer are warranted to identify whether direct or indirect (through weight gain or overweight or obesity status, which are strong risk factors for multiple cancers^41^) associations exist for various cancer types.

In addition, we observed that a higher level of total soft drink consumption was associated with greater risk of Parkinson disease mortality, with positive nonsignificant associations found for sugar-sweetened and artificially sweetened soft drinks. To our knowledge, this study is the first to link soft drink consumption with Parkinson disease, and additional studies are required to examine this association.

### Limitations

To our knowledge, this study is the largest to date to investigate the association between soft drink consumption and mortality. However, it has several limitations. Given the observational design of the study, it is not possible to establish causality between soft drink consumption and mortality, and we recognize that the observed associations may be biased because of residual confounding. However, the large number of participants and recorded deaths (approximately 42 000) allowed us to conduct analyses by subgroups of other mortality risk factors, and we generally observed similar associations across subgroups of considered risk factors. Furthermore, the negative control analysis found no association between consumption of soft drinks and deaths from external causes. This study was also limited by a single assessment of soft drink consumption at baseline.

## Conclusions

In this study, the high level of consumption of total, sugar-sweetened, and artificially sweetened soft drinks was associated with elevated risks of death from all causes. Positive associations were observed between sugar-sweetened soft drinks and digestive disease deaths as well as between artificially sweetened soft drinks and circulatory disease deaths. Further studies are needed to investigate the possible adverse health effects of artificial sweeteners. The results of this study are supportive of ongoing public health campaigns aimed at reducing the consumption of soft drinks.

## Notes

Supplement. **eFigure.** Hazard Ratio (HR) Functions and Corresponding 95% CIs Describing the Linear (Light Blue) and the Curve-Linear (Dark Blue) Dose–Response Relationship Between Soft Drinks Consumption (mL/d) and All-Cause Mortality Risk, According to Death Frequencies **eTable 1.** Analysis of Association Between Soft Drinks Consumption and All-Cause Mortality by Country **eTable 2.** Body Mass Index Subgroup Analysis of Association Between Artificially Sweetened and Sugar-Sweetened Soft Drinks Consumption and All-Cause and Cause-Specific Mortality **eTable 3.** Multivariable Associations of Categories of Soft Drinks Consumption and All-Cause and Cause-Specific Mortality With and Without Adjustment for Body Mass Index (Sexes Combined) **eTable 4.** Multivariable Associations of Categories of Soft Drinks Consumption and All-Cause and Cause-Specific Mortality With Deaths That Occurred During the First 8 Years of Follow-up Excluded **eTable 5.** Multivariable Associations of Categories of Soft Drinks Consumption and All-Cause and Cause-Specific Mortality With Non-Consumers as the Reference Group **eTable 6.** Multivariable Associations of Categories of Soft Drinks Consumption and All-Cause and Cause-Specific Mortality With Adjustment for an Adapted Version of the WCRF Dietary Score (Rather than Individual Dietary Covariates) **eTable 7.** Associations of Categories of Artificially Sweetened and Sugar-Sweetened Soft Drinks Consumption With All-Cause and Cause-Specific Mortality Among Participants Who Solely Consumed Sugar-Sweetened or Artificially Sweetened Soft Drinks **eTable 8.** Multivariable Associations of Categories of Soft Drinks Consumption and All-Cause Mortality by Ascertainment of Death Method **eTable 9.** Multivariable Associations of Categories of Soft Drinks Consumption and Deaths Due to External Causes (ICD-10 Codes S00-Y98)
