# Supplementary material for: Association Between Soft Drink Consumption and Mortality in 10 European Countries
Source: JAMA Intern Med. 2019 Sep 3;179(11):1479–90. doi: 10.1001/jamainternmed.2019.2478 (PMC6724165; doi:10.1001/jamainternmed.2019.2478)
Supplement: Supplement. — eFigure. Hazard Ratio (HR) Functions and Corresponding 95% CIs Describing the Linear (Light Blue) and the Curve-Linear (Dark Blue) Dose–Response Relationship Between Soft Drinks Consumption (mL/d) and All-Cause Mortality Risk, According to Death Frequencies eTable 1. Analysis of Association Between Soft Drinks Consumption and All-Cause Mortality by Country eTable 2. Body Mass Index Subgroup Analysis of Association Between Artificially Sweetened and Sugar-Sweetened Soft Drinks Consumption and All-Cause and Cause-Specific Mortality eTable 3. Multivariable Associations of Categories of Soft Drinks Consumption and All-Cause and Cause-Specific Mortality With and Without Adjustment for Body Mass Index (Sexes Combined) eTable 4. Multivariable Associations of Categories of Soft Drinks Consumption and All-Cause and Cause-Specific Mortality With Deaths That Occurred During the First 8 Years of Follow-up Excluded eTable 5. Multivariable Associations of Categories of Soft Drinks Consumption and All-Cause and Cause-Specific Mortality With Non-Consumers as the Reference Group eTable 6. Multivariable Associations of Categories of Soft Drinks Consumption and All-Cause and Cause-Specific Mortality With Adjustment for an Adapted Version of the WCRF Dietary Score (Rather than Individual Dietary Covariates) eTable 7. Associations of Categories of Artificially Sweetened and Sugar-Sweetened Soft Drinks Consumption With All-Cause and Cause-Specific Mortality Among Participants Who Solely Consumed Sugar-Sweetened or Artificially Sweetened Soft Drinks eTable 8. Multivariable Associations of Categories of Soft Drinks Consumption and All-Cause Mortality by Ascertainment of Death Method eTable 9. Multivariable Associations of Categories of Soft Drinks Consumption and Deaths Due to External Causes (ICD-10 Codes S00-Y98) [file jamainternmed-e192478-s001.pdf]

Supplementary Online Content

Mullee A, Romaguera D, Pearson-Stuttard J, et al. Association between soft drink consumption and mortality in 10 European countries. *JAMA Intern Med.* Published online September 3, 2019. doi:10.1001/jamainternmed.2019.2478

eFigure. Hazard Ratio (HR) Functions and Corresponding 95% CIs Describing the Linear (Light Blue) and the Curve-Linear (Dark Blue) Dose–Response Relationship Between Soft Drinks Consumption (mL/day) and All-Cause Mortality Risk, According to Death Frequencies

eTable 1. Analysis of Association Between Soft Drinks Consumption and All-Cause Mortality by Country

eTable 2. Body Mass Index Subgroup Analysis of Association Between Artificially Sweetened and Sugar-Sweetened Soft Drinks Consumption and All-Cause and Cause-Specific Mortality

eTable 3. Multivariable Associations of Categories of Soft Drinks Consumption and All-Cause and Cause-Specific Mortality With and Without Adjustment for Body Mass Index (Sexes Combined)

eTable 4. Multivariable Associations of Categories of Soft Drinks Consumption and All-Cause and Cause-Specific Mortality With Deaths That Occurred During the First 8 Years of Follow-up Excluded

eTable 5. Multivariable Associations of Categories of Soft Drinks Consumption and All-Cause and Cause-Specific Mortality With Non-Consumers as the Reference Group

eTable 6. Multivariable Associations of Categories of Soft Drinks Consumption and All-Cause and Cause-Specific Mortality With Adjustment for an Adapted Version of the WCRF Dietary Score (Rather than Individual Dietary Covariates)

eTable 7. Associations of Categories of Artificially Sweetened and Sugar-Sweetened Soft Drinks Consumption With All-Cause and Cause-Specific Mortality Among Participants Who Solely Consumed Sugar-Sweetened or Artificially Sweetened Soft Drinks

eTable 8. Multivariable Associations of Categories of Soft Drinks Consumption and All-Cause Mortality by Ascertainment of Death Method

eTable 9. Multivariable Associations of Categories of Soft Drinks Consumption and Deaths Due to External Causes (ICD-10 Codes S00-Y98)

This supplementary material has been provided by the authors to give readers additional information about their work.

© 2019 Mullee A et al. *JAMA Internal Medicine.*

eFigure. Hazard Ratio (HR) Functions and Corresponding 95% CIs Describing the Linear (Light Blue) and the Curve-Linear (Dark Blue) Dose–Response Relationship Between Soft Drinks Consumption (mL/day) and All-Cause Mortality Risk, According to Death Frequencies

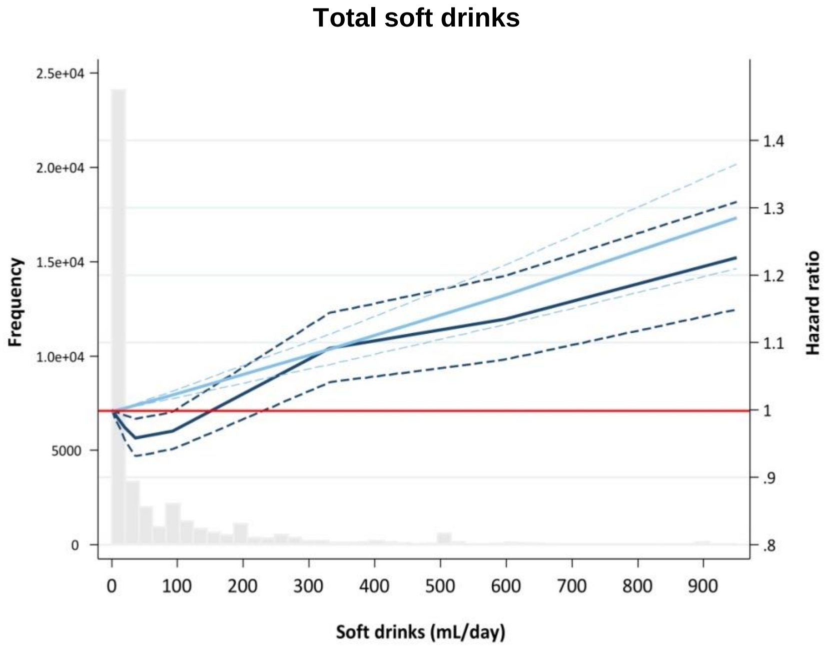

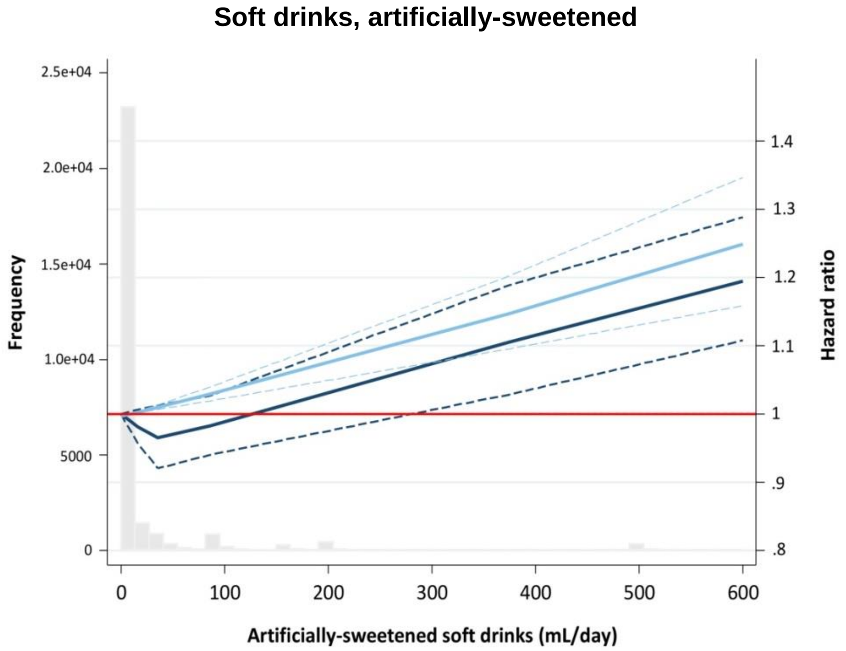

© 2019 Mullee A et al. *JAMA Internal Medicine.*

Soft drinks, sugar-sweetened

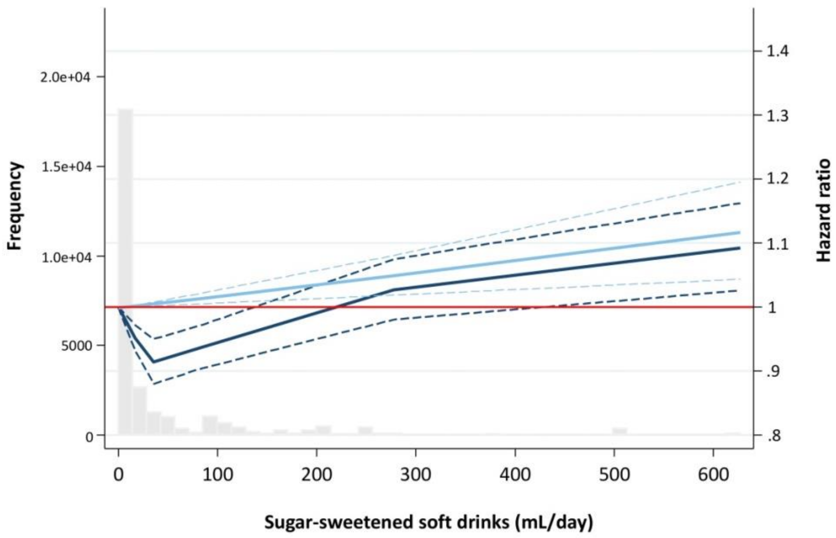

Multivariable model-Cox regression using body mass index (<22; 22-<25; 25-<30; 30-<35; ≥35 kg/m2), physical activity index (inactive; moderately inactive; moderately active; active), education status (none; primary school completed; technical/professional school; secondary school; longer education including university; not specified), alcohol consumption (non-consumers; <5; 5-<15; 15-<30; ≥30 g/day), smoking status and intensity (never; current , 1-15 cigarettes per day; current, 16-25 cigarettes per day; current, >25 cigarettes per day; former, quit ≤10 years; former, quit 11-20 years; former, quit >20 years; current, pipe/cigar/occasional; current/former, missing; unknown), smoking duration (<10; 10-<20; 20-<30; 30-<40; ≥40 years; smoking duration unknown), menopausal status (premenopausal; postmenopausal; perimenopausal/unknown menopausal status; surgical postmenopausal), ever use of menopausal hormone therapy (yes; no; unknown), and intakes of total energy (kcal/day), red and processed meat (g/day), fruits and vegetables (g/day), coffee (g/day), and juice (g/day) (all continuous), and stratified by age (1-year categories), center, and sex. Additionally, sugar sweetened and artificially sweetened soft drinks were mutually adjusted. Italy, Spain and Sweden are excluded from the sugar-sweetened and artificially-sweetened soft drinks analyses as information on type of soft drink consumption was not collected.

© 2019 Mullee A et al. *JAMA Internal Medicine.*

eTable 1. Analysis of Association Between Soft Drinks Consumption and All-Cause Mortality by Country

Country	Total soft drinks	Soft drinks, sugar sweetened	Soft drinks, artificially-sweetened
Denmark			
N	51,193	51,193	51,193
Multivariable model, HR (95% CI)	1.05 (0.98-1.13)	0.92 (0.82-1.06)	1.20 (1.10-1.32)
France			
N	65,566	65,566	65,566
Multivariable model, HR (95% CI)	1.50 (1.07-2.10)	1.70 (1.02-2.84)	1.38 (0.80-2.38)
Germany			
N	45,432	45,432	45,432
Multivariable model, HR (95% CI)	1.19 (1.04-1.37)	1.21 (1.03-1.42)	1.11 (0.88-1.40)
Greece			
N	23,014	23,014	23,014
Multivariable model, HR (95% CI)	1.29 (1.04-1.61)	1.60 (1.21-2.12)	1.41 (0.51-3.90)
Italy			
N	42,945	-	-
Multivariable model, HR (95% CI)	1.38 (1.11-1.71)	-	-
The Netherlands			
N	35,069	35,069	35,069
Multivariable model, HR (95% CI)	1.05 (0.92-1.19)	1.16 (0.99-1.37)	1.00 (0.72-1.40)
Norway			
N	33,341	33,341	33,341
Multivariable model, HR (95% CI)	1.10 (0.89-1.36)	1.21 (0.90-1.63)	1.05 (0.82-1.36)
Spain			
N	37,321	-	-

© 2019 Mullee A et al. *JAMA Internal Medicine.*

Multivariable model, HR (95% CI)	1.16 (0.96-1.40)	-	-
Sweden			
N	46,497	-	-
Multivariable model, HR (95% CI)	1.15 (1.07-1.25)	-	-
United Kingdom			
N	71,365	71,365	71,365
Multivariable model, HR (95% CI)	1.09 (1.01-1.17)	1.06 (0.98-1.15)	1.19 (1.03-1.38)
**Random effects meta-analysis**	1.11 (1.07-1.15)	1.07 (1.01-1.13)	1.17 (1.09-1.25)
**I^2**	26%	63%	0%
***p*-heterogeneity**	0.21	0.01	0.86
**Cox proportional hazards models stratified by center**	1.13 (1.09-1.17)	1.08 (1.03-1.14)	1.20 (1.12-1.29)

HR = hazard ratio; CI = confidence interval. HRs for the comparison of participants drinking ≥1 glass per day versus <1 glass per month. Multivariable model -Cox regression using body mass index (<22; 22-<25; 25-<30; 30-<35; ≥35 kg/m^2), physical activity index (inactive; moderately inactive; moderately active; active), education status (none; primary school completed; technical/professional school; secondary school; longer education including university; or not specified), alcohol consumption (non-consumers; <5; 5-<15; 15-<30; ≥30 g/day), smoking status and intensity (never; current , 1-15 cigarettes per day; current, 16-25 cigarettes per day; current, ≥16 cigarettes per day; former, quit ≤10 years; former, quit 11-20 years; former, quit ≥20 years; current, pipe/cigar/occasional; current/former, missing; unknown), smoking duration (<10; 10-<20; 20-<30; 30-<40; ≥40 years; smoking duration unknown), ever use of contraceptive pill (yes; no; or unknown), menopausal status (premenopausal; postmenopausal; perimenopausal/unknown menopausal status; or surgical postmenopausal), ever use of menopausal hormone therapy (yes; no; or unknown), and intakes of total energy (kcal/day), red and processed meat (g/day), fruits and vegetables (g/day), coffee (g/day), and fruit and vegetable juice (g/day) (all continuous), and stratified by age (1-year categories), center, and sex.

© 2019 Mullee A et al. *JAMA Internal Medicine.*

eTable 2. Body Mass Index Subgroup Analysis of Association Between Artificially Sweetened and Sugar-Sweetened Soft Drinks Consumption and All-Cause and Cause-Specific Mortality

	Soft drinks, artificially sweetened	*p-interaction*		Soft drinks, sugar sweetened	*p-interaction*
	**Multivariable model, HR (95% CI)**			**Multivariable model, HR (95% CI)**	
**All-cause mortality**					
Body mass index, kg/m^2		0.54			0.002
<25	1.27 (1.12-1.43)			1.11 (1.03-1.21)	
25-<30	1.16 (1.04-1.29)			0.98 (0.90-1.06)	
≥30	1.22 (1.07-1.39)			1.23 (1.10-1.39)	
**Cancer (ICD-10 codes C00-D48)**					
Body mass index, kg/m^2		0.33			0.28
<25	1.09 (0.91-1.30)			0.94 (0.82-1.07)	
25-<30	1.07 (0.91-1.26)			0.92 (0.80-1.05)	
≥30	1.02 (0.82-1.27)			1.09 (0.89-1.33)	
**Circulatory diseases (ICD-10 codes I00-I99)**					
Body mass index, kg/m^2		0.48			0.07
<25	1.59 (1.19-2.13)			1.20 (1.00-1.44)	
25-<30	1.22 (0.96-1.54)			0.90 (0.76-1.07)	
≥30	1.59 (1.23-2.06)			1.33 (1.06-1.67)	
**Digestive diseases (ICD-10 codes K00-K93)**					
Body mass index, kg/m^2		0.03			0.96
<25	2.38 (1.31-4.34)			1.80 (1.18-2.74)	
25-<30	0.84 (0.42-1.67)			1.48 (0.99-2.21)	
≥30	0.29 (0.09-0.92)			1.42 (0.82-2.46)	

© 2019 Mullee A et al. *JAMA Internal Medicine.*

HR = hazard ratio; CI = confidence interval. HRs for the comparison of participants drinking ≥1 glass per day versus <1 glass per month. ICD-10 = International Classification of Diseases, 10th Revision. Multivariate model-Cox regression using body mass index (<22; 22-<25; 25-<30; 30-<35; 35+ kg/m^2), physical activity index (inactive; moderately inactive; moderately active; active), education status (none; primary school completed; technical/professional school; secondary school; longer education including university; or not specified), alcohol consumption (non-consumers; <5; 5-<15; 15-<30; 30+ g/day), smoking status and intensity (never; current , 1-15 cigarettes per day; current, 16-25 cigarettes per day; current, 16+ cigarettes per day; former, quit ≤10 years; former, quit 11-20 years; former, quit 20+ years; current, pipe/cigar/occasional; current/former, missing; unknown), smoking duration (<10; 10-<20; 20-<30; 30-<40; 40+ years; smoking duration unknown), ever use of contraceptive pill (yes; no; or unknown), menopausal status (premenopausal; postmenopausal; perimenopausal/unknown menopausal status; or surgical postmenopausal), ever use of menopausal hormone therapy (yes; no; or unknown), and intakes of total energy (kcal/day), red and processed meat (g/day), and fruits and vegetables (g/day), coffee (g/day), juice (g/day) (all continuous), and stratified by age (1-year categories), and center.

© 2019 Mullee A et al. *JAMA Internal Medicine.*

**eTable 3.** Multivariable Associations of Categories of Soft Drinks Consumption and All-Cause and Cause-Specific Mortality With and Without Adjustment for Body Mass Index (Sexes Combined)						
	**<1 glass/month**	**1–4 glasses/month**	**>1–6 glasses/week**	**1-<2 glasses/day**	**≥2 glasses/day**	***ptrend***
**All-cause mortality**						
**Total soft drinks**						
Multivariable model, HR (95% CI)	1 (ref)	0.97 (0.94-1.00)	0.98 (0.96-1.01)	1.10 (1.06-1.16)	1.17 (1.11-1.22)	<.0001
Multivariable model excluding body mass index, HR (95% CI)	1 (ref)	0.97 (0.94-1.00)	0.99 (0.96-1.02)	1.12 (1.07-1.17)	1.19 (1.13-1.25)	<.0001
**Soft drinks, artificially-sweetened**						
Multivariable model, HR (95% CI)	1 (ref)	0.93 (0.89-0.97)	1.01 (0.97-1.05)	0.99 (0.84-1.17)	1.26 (1.16-1.35)	<.0001
Multivariable model excluding body mass index, HR (95% CI)	1 (ref)	0.94 (0.90-0.98)	1.02 (0.98-1.07)	1.03 (0.87-1.21)	1.30 (1.21-1.40)	<.0001
**Soft drinks, sugar-sweetened**						
Multivariable model, HR (95% CI)	1 (ref)	0.94 (0.91-0.98)	0.96 (0.93-1.00)	1.08 (1.01-1.16)	1.08 (1.01-1.16)	0.004
Multivariable model excluding body mass index, HR (95% CI)	1 (ref)	0.94 (0.91-0.98)	0.97 (0.93-1.00)	1.08 (1.01-1.15)	1.09 (1.01-1.17)	0.003
**Cancer (ICD-10 codes C00-D48)**						
**Total soft drinks**						
Multivariable model, HR (95% CI)	1 (ref)	0.99 (0.95-1.04)	1.00 (0.96-1.04)	1.02 (0.95-1.10)	1.02 (0.95-1.11)	0.45
Multivariable model excluding body mass index, HR (95% CI)	1 (ref)	1.00 (0.95-1.04)	1.00 (0.97-1.04)	1.03 (0.95-1.10)	1.03 (0.96-1.12)	0.29
**Soft drinks, artificially-sweetened**						
Multivariable model, HR (95% CI)	1 (ref)	0.96 (0.90-1.02)	1.00 (0.94-1.06)	0.92 (0.73-1.16)	1.10 (0.97-1.23)	0.23
Multivariable model excluding body mass index, HR (95% CI)	1 (ref)	0.96 (0.90-1.02)	1.01 (0.95-1.07)	0.93 (0.74-1.18)	1.11 (0.99-1.25)	0.14
**Soft drinks, sugar-sweetened**						
Multivariable model, HR (95% CI)	1 (ref)	0.95 (0.90-1.01)	0.97 (0.92-1.02)	0.97 (0.86-1.09)	0.95 (0.84-1.06)	0.33
Multivariable model excluding body mass index, HR (95% CI)	1 (ref)	0.95 (0.90-1.01)	0.97 (0.92-1.02)	0.97 (0.86-1.09)	0.95 (0.85-1.06)	0.35
**Circulatory diseases (ICD-10 codes I00-I99)**						
**Total soft drinks**						

© 2019 Mullee A et al. *JAMA Internal Medicine.*

Multivariable model, HR (95% CI)	1 (ref)	0.96 (0.90-1.02)	0.98 (0.93-1.03)	1.19 (1.09-1.31)	1.27 (1.14-1.40)	<.0001
Multivariable model excluding body mass index, HR (95% CI)	1 (ref)	0.97 (0.91-1.03)	1.00 (0.95-1.06)	1.23 (1.13-1.35)	1.33 (1.20-1.48)	<.0001
**Soft drinks, artificially-sweetened**						
Multivariable model, HR (95% CI)	1 (ref)	0.91 (0.83-1.00)	1.01 (0.92-1.11)	1.02 (0.70-1.50)	1.52 (1.30-1.78)	<.0001
Multivariable model excluding body mass index, HR (95% CI)	1 (ref)	0.92 (0.84-1.02)	1.07 (0.97-1.17)	1.11 (0.76-1.63)	1.65 (1.41-1.93)	<.0001
**Soft drinks, sugar-sweetened**						
Multivariable model, HR (95% CI)	1 (ref)	0.97 (0.90-1.05)	0.96 (0.90-1.04)	1.06 (0.92-1.22)	1.11 (0.95-1.30)	0.16
Multivariable model excluding body mass index, HR (95% CI)	1 (ref)	0.98 (0.91-1.05)	0.97 (0.91-1.05)	1.07 (0.93-1.23)	1.13 (0.96-1.32)	0.10
**Digestive diseases (ICD-10 codes K00-K93)‡**						
**Total soft drinks**						
Multivariable model, HR (95% CI)	1 (ref)	1.07 (0.89-1.28)	1.16 (1.00-1.34)	1.50 (1.24-1.81)		<.0001
Multivariable model excluding body mass index, HR (95% CI)	1 (ref)	1.08 (0.90-1.29)	1.18 (1.02-1.37)	1.56 (1.29-1.88)		<.0001
**Soft drinks, artificially-sweetened**						
Multivariable model, HR (95% CI)	1 (ref)	1.00 (0.79-1.27)	1.19 (0.95-1.50)	0.99 (0.65-1.50)		0.78
Multivariable model excluding body mass index, HR (95% CI)	1 (ref)	1.02 (0.81-1.29)	1.27 (1.01-1.59)	1.10 (0.72-1.66)		0.39
**Soft drinks, sugar-sweetened**						
Multivariable model, HR (95% CI)	1 (ref)	1.05 (0.86-1.28)	1.07 (0.88-1.29)	1.59 (1.24-2.05)		<.0001
Multivariable model excluding body mass index, HR (95% CI)	1 (ref)	1.05 (0.86-1.28)	1.07 (0.88-1.30)	1.60 (1.24-2.06)		<.0001

HR = hazard ratio; CI = confidence interval. ICD-10 = International Classification of Diseases, 10th Revision. Multivariable model-Cox regression using body mass index (<22; 22-<25; 25-<30; 30-<35; ≥35 kg/m^2), physical activity index (inactive; moderately inactive; moderately active; active), education status (none; primary school completed; technical/professional school; secondary school; longer education including university; or not specified), alcohol consumption (non-consumers; <5; 5-<15; 15-<30; ≥30 g/day), smoking status and intensity (never; current , 1-15 cigarettes per day; current, 16-25 cigarettes per day; current, ≥16 cigarettes per day; former, quit ≤10 years; former, quit 11-20 years; former, quit ≥20 years; current, pipe/cigar/occasional; current/former, missing; unknown), smoking duration (<10; 10-<20; 20-<30; 30-<40; ≥40 years; smoking duration unknown), ever use of contraceptive pill (yes; no; or unknown), menopausal status (premenopausal; postmenopausal; perimenopausal/unknown menopausal status; or surgical postmenopausal), ever use of menopausal hormone therapy (yes; no; or unknown), and intakes of total energy (kcal/day), red and processed meat (g/day), fruits and vegetables (g/day), coffee (g/day), and fruit and vegetable juice (g/day) (all continuous), and stratified by age (1-year categories), center, and sex. Additionally, sugar sweetened and artificially sweetened soft drinks were mutually adjusted. Italy, Spain and Sweden are excluded from the sugar-sweetened and artificially-sweetened soft drinks analyses as information on type of soft drink consumption was not collected. ‡Top two categories were merged as '≥1 glasses/day' due to low case numbers.

© 2019 Mullee A et al. *JAMA Internal Medicine.*

eTable 4. Multivariable Associations of Categories of Soft Drinks Consumption and All-Cause and Cause-Specific Mortality With Deaths That Occurred During the First 8 Years of Follow-up Excluded

	**<1 glass/month**	**1–4 glasses/month**	**>1–6 glasses/week**	**1-<2 glasses/day**	**≥2 glasses/day**	***ptrend***
**All-cause mortality**						
Total soft drinks, HR (95% CI)	1 (ref)	0.95 (0.92-0.99)	0.98 (0.95-1.01)	1.09 (1.04-1.15)	1.14 (1.07-1.21)	<.0001
Soft drinks, artificially-sweetened, HR (95% CI)	1 (ref)	0.93 (0.89-0.98)	0.98 (0.94-1.03)	0.98 (0.80-1.19)	1.24 (1.14-1.36)	<.0001
Soft drinks, sugar-sweetened, HR (95% CI)	1 (ref)	0.93 (0.89-0.97)	0.96 (0.92-1.00)	1.06 (0.98-1.15)	1.04 (0.95-1.13)	0.12
**Cancer (ICD-10 codes C00-D48)**						
Total soft drinks, HR (95% CI)	1 (ref)	0.98 (0.93-1.04)	1.01 (0.96-1.05)	1.00 (0.91-1.09)	1.03 (0.94-1.14)	0.47
Soft drinks, artificially-sweetened, HR (95% CI)	1 (ref)	0.95 (0.88-1.03)	1.00 (0.93-1.08)	0.91 (0.69-1.22)	1.12 (0.97-1.29)	0.20
Soft drinks, sugar-sweetened, HR (95% CI)	1 (ref)	0.93 (0.88-1.00)	0.96 (0.90-1.02)	0.93 (0.81-1.08)	0.92 (0.80-1.06)	0.19
**Circulatory diseases (ICD-10 codes I00-I99)**						
Total soft drinks, HR (95% CI)	1 (ref)	0.93 (0.86-1.01)	0.97 (0.91-1.04)	1.19 (1.06-1.32)	1.22 (1.07-1.38)	<.0001
Soft drinks, artificially-sweetened, HR (95% CI)	1 (ref)	0.86 (0.76-0.96)	0.94 (0.83-1.05)	0.91 (0.55-1.49)	1.44 (1.18-1.76)	0.002
Soft drinks, sugar-sweetened, HR (95% CI)	1 (ref)	0.97 (0.88-1.06)	0.96 (0.88-1.05)	1.13 (0.95-1.35)	1.08 (0.88-1.31)	0.25
**Digestive diseases (ICD-10 codes K00-K93)‡**						
Total soft drinks, HR (95% CI)	1 (ref)	1.05 (0.85-1.30)	1.17 (0.99-1.39)	1.39 (1.10-1.75)		0.004

© 2019 Mullee A et al. *JAMA Internal Medicine.*

Soft drinks, artificially-sweetened, HR (95% CI)	1 (ref)	1.15 (0.88-1.49)	1.11 (0.84-1.47)	0.86 (0.50-1.47)	0.66
Soft drinks, sugar-sweetened, HR (95% CI)	1 (ref)	1.06 (0.84-1.34)	1.11 (0.88-1.39)	1.36 (0.99-1.88)	0.06

HR = hazard ratio; CI = confidence interval. ICD-10 = International Classification of Diseases, 10th Revision. Multivariable model-Cox regression using body mass index (<22; 22-<25; 25-<30; 30-<35; ≥35 kg/m^2), physical activity index (inactive; moderately inactive; moderately active; active), education status (none; primary school completed; technical/professional school; secondary school; longer education including university; or not specified), alcohol consumption (non-consumers; <5; 5-<15; 15-<30; ≥30 g/day), smoking status and intensity (never; current , 1-15 cigarettes per day; current, 16-25 cigarettes per day; current, ≥16 cigarettes per day; former, quit ≤10 years; former, quit 11-20 years; former, quit ≥20 years; current, pipe/cigar/occasional; current/former, missing; unknown), smoking duration (<10; 10-<20; 20-<30; 30-<40; ≥40 years; smoking duration unknown), ever use of contraceptive pill (yes; no; or unknown), menopausal status (premenopausal; postmenopausal; perimenopausal/unknown menopausal status; or surgical postmenopausal), ever use of menopausal hormone therapy (yes; no; or unknown), and intakes of total energy (kcal/day), red and processed meat (g/day), fruits and vegetables (g/day), coffee (g/day), and fruit and vegetable juice (g/day) (all continuous), and stratified by age (1-year categories), center, and sex. Additionally, sugar sweetened and artificially sweetened soft drinks were mutually adjusted. Italy, Spain and Sweden are excluded from the sugar-sweetened and artificially-sweetened soft drinks analyses as information on type of soft drink consumption was not collected. ‡Top two categories were merged as '≥1 glasses/day' due to low case numbers.

© 2019 Mullee A et al. *JAMA Internal Medicine.*

eTable 5. Multivariable Associations of Categories of Soft Drinks Consumption and All-Cause and Cause-Specific Mortality With Non-Consumers as the Reference Group

	**Non-consumers**	**<1 glass/month**	**1–4 glasses/month**	**>1–6 glasses/week**	**1-<2 glasses/day**	**≥2 glasses/day**	***ptrend***
**All-cause mortality**							
Total soft drinks, HR (95% CI)	1 (ref)	0.95 (0.91-0.99)	0.95 (0.92-0.98)	0.97 (0.94-1.00)	1.09 (1.04-1.14)	1.15 (1.09-1.21)	<.0001
Soft drinks, artificially-sweetened, HR (95% CI)	1 (ref)	1.01 (0.96-1.05)	0.94 (0.90-0.98)	1.01 (0.97-1.06)	0.99 (0.84-1.17)	1.26 (1.17-1.36)	<.0001
Soft drinks, sugar-sweetened, HR (95% CI)	1 (ref)	0.92 (0.88-0.96)	0.92 (0.88-0.95)	0.94 (0.91-0.97)	1.05 (0.98-1.13)	1.05 (0.98-1.13)	0.005
**Cancer (ICD-10 codes C00-D48)**							
Total soft drinks, HR (95% CI)	1 (ref)	1.01 (0.95-1.07)	1.00 (0.95-1.05)	1.00 (0.96-1.05)	1.02 (0.95-1.10)	1.02 (0.95-1.11)	0.44
Soft drinks, artificially-sweetened, HR (95% CI)	1 (ref)	1.05 (0.98-1.12)	0.98 (0.91-1.04)	1.02 (0.95-1.08)	0.93 (0.73-1.17)	1.11 (0.99-1.25)	0.22
Soft drinks, sugar-sweetened, HR (95% CI)	1 (ref)	0.98 (0.92-1.04)	0.95 (0.89-1.00)	0.96 (0.91-1.01)	0.96 (0.86-1.08)	0.94 (0.84-1.06)	0.33
**Circulatory diseases (ICD-10 codes I00-I99)**							
Total soft drinks, HR (95% CI)	1 (ref)	0.94 (0.87-1.03)	0.94 (0.88-1.01)	0.96 (0.91-1.02)	1.18 (1.07-1.29)	1.25 (1.12-1.39)	<.0001
Soft drinks, artificially-sweetened, HR (95% CI)	1 (ref)	1.04 (0.95-1.15)	0.92 (0.83-1.02)	1.02 (0.93-1.13)	1.03 (0.70-1.51)	1.54 (1.31-1.81)	<.0001
Soft drinks, sugar-sweetened, HR (95% CI)	1 (ref)	0.86 (0.78-0.94)	0.92 (0.85-1.00)	0.91 (0.85-0.99)	1.00 (0.87-1.16)	1.06 (0.90-1.24)	0.17

© 2019 Mullee A et al. *JAMA Internal Medicine.*

HR = hazard ratio; CI = confidence interval. Multivariable model-Cox regression using body mass index (<22; 22-<25; 25-<30; 30-<35; ≥35 kg/m^2), physical activity index (inactive; moderately inactive; moderately active; active), education status (none; primary school completed; technical/professional school; secondary school; longer education including university; or not specified), alcohol consumption (non-consumers; <5; 5-<15; 15-<30; ≥30 g/day), smoking status and intensity (never; current , 1-15 cigarettes per day; current, 16-25 cigarettes per day; current, ≥16 cigarettes per day; former, quit ≤10 years; former, quit 11-20 years; former, quit ≥20 years; current, pipe/cigar/occasional; current/former, missing; unknown), smoking duration (<10; 10-<20; 20-<30; 30-<40; ≥40 years; smoking duration unknown), ever use of contraceptive pill (yes; no; or unknown), menopausal status (premenopausal; postmenopausal; perimenopausal/unknown menopausal status; or surgical postmenopausal), ever use of menopausal hormone therapy (yes; no; or unknown), and intakes of total energy (kcal/day), red and processed meat (g/day), fruits and vegetables (g/day), coffee (g/day), and fruit and vegetable juice (g/day) (all continuous), and stratified by age (1-year categories), center, and sex. Additionally, sugar sweetened and artificially sweetened soft drinks were mutually adjusted. Italy, Spain and Sweden are excluded from the sugar-sweetened and artificially-sweetened soft drinks analyses as information on type of soft drink consumption was not collected.

© 2019 Mullee A et al. *JAMA Internal Medicine.*

eTable 6. Multivariable Associations of Categories of Soft Drinks Consumption and All-Cause and Cause-Specific Mortality With Adjustment for an Adapted Version of the WCRF Dietary Score (Rather than Individual Dietary Covariates)

	<1 glass/month	1–4 glasses/month	>1–6 glasses/week	1-<2 glasses/day	≥2 glasses/day	*ptrend*
**All-cause mortality**						
Total soft drinks, HR (95% CI)	1 (ref)	0.96 (0.93-0.99)	0.97 (0.95-1.00)	1.08 (1.03-1.13)	1.14 (1.08-1.19)	<.0001
Soft drinks, artificially-sweetened, HR (95% CI)	1 (ref)	0.93 (0.90-0.97)	1.01 (0.96-1.05)	0.99 (0.84-1.16)	1.24 (1.15-1.34)	<.0001
Soft drinks, sugar-sweetened, HR (95% CI)	1 (ref)	0.94 (0.90-0.97)	0.95 (0.92-0.99)	1.06 (0.99-1.13)	1.06 (0.99-1.14)	0.03
**Cancer (ICD-10 codes C00-D48)**						
Total soft drinks, HR (95% CI)	1 (ref)	0.99 (0.94-1.03)	0.99 (0.95-1.03)	0.99 (0.92-1.06)	0.99 (0.92-1.08)	0.86
Soft drinks, artificially-sweetened, HR (95% CI)	1 (ref)	0.96 (0.90-1.02)	0.99 (0.93-1.06)	0.90 (0.72-1.14)	1.08 (0.96-1.22)	0.35
Soft drinks, sugar-sweetened, HR (95% CI)	1 (ref)	0.95 (0.90-1.00)	0.95 (0.91-1.00)	0.94 (0.84-1.06)	0.93 (0.83-1.04)	0.14
**Circulatory diseases (ICD-10 codes I00-I99)**						
Total soft drinks, HR (95% CI)	1 (ref)	0.95 (0.89-1.01)	0.97 (0.92-1.02)	1.16 (1.06-1.27)	1.23 (1.11-1.37)	<.0001
Soft drinks, artificially-sweetened, HR (95% CI)	1 (ref)	0.91 (0.83-1.00)	1.00 (0.91-1.10)	1.01 (0.69-1.48)	1.49 (1.28-1.75)	<.0001
Soft drinks, sugar-sweetened, HR (95% CI)	1 (ref)	0.96 (0.89-1.04)	0.95 (0.88-1.02)	1.03 (0.90-1.19)	1.08 (0.93-1.27)	0.29
**Digestive diseases (ICD-10 codes K00-K93)**						
Total soft drinks, HR (95% CI)	1 (ref)	1.04 (0.87-1.24)	1.13 (0.97-1.31)	1.43 (1.18-1.74)		<.0001
Soft drinks, artificially-sweetened, HR (95% CI)	1 (ref)	0.99 (0.79-1.25)	1.16 (0.92-1.46)	0.94 (0.62-1.43)		0.99
Soft drinks, sugar-sweetened, HR (95% CI)	1 (ref)	1.03 (0.84-1.25)	1.05 (0.86-1.27)	1.54 (1.19-1.98)		0.001

© 2019 Mullee A et al. *JAMA Internal Medicine.*

HR = hazard ratio; CI = confidence interval. Multivariable model-Cox regression using body mass index (<22; 22-<25; 25-<30; 30-<35; ≥35 kg/m^2), physical activity index (inactive; moderately inactive; moderately active; active), education status (none; primary school completed; technical/professional school; secondary school; longer education including university; or not specified), alcohol consumption (non-consumers; <5; 5-<15; 15-<30; ≥30 g/day), smoking status and intensity (never; current , 1-15 cigarettes per day; current, 16-25 cigarettes per day; current, ≥16 cigarettes per day; former, quit ≤10 years; former, quit 11-20 years; former, quit ≥20 years; current, pipe/cigar/occasional; current/former, missing; unknown), smoking duration (<10; 10-<20; 20-<30; 30-<40; ≥40 years; smoking duration unknown), ever use of contraceptive pill (yes; no; or unknown), menopausal status (premenopausal; postmenopausal; perimenopausal/unknown menopausal status; or surgical postmenopausal), ever use of menopausal hormone therapy (yes; no; or unknown), intake of total energy (kcal/day), WCRF dietary score (sex-specific quintiles), and stratified by age (1-year categories), center, and sex. Italy, Spain and Sweden are excluded from the sugar-sweetened and artificially-sweetened soft drinks analyses as information on type of soft drink consumption was not collected.

© 2019 Mullee A et al. *JAMA Internal Medicine.*

eTable 7. Associations of Categories of Artificially Sweetened and Sugar-Sweetened Soft Drinks Consumption With All-Cause and Cause-Specific Mortality Among Participants Who Solely Consumed Sugar-Sweetened or Artificially Sweetened Soft Drinks

	**<1 glass/month**	**1–4 glasses/month**	**>1–6 glasses/week**	**≥1 glass/day**	***ptrend***
**All-cause mortality**					
**Soft drinks, artificially sweetened**					
Deaths, n	11141	600	997	530	
Multivariable model, HR (95% CI)	1 (ref)	0.92 (0.85-1.00)	1.04 (0.98-1.12)	1.22 (1.11-1.33)	<.0001
**Soft drinks, sugar sweetened**					
Deaths, n	11866	2144	2696	999	
Multivariable model, HR (95% CI)	1 (ref)	0.95 (0.90-1.00)	0.97 (0.92-1.01)	1.11 (1.04-1.19)	0.003
**Cancer (ICD-10 codes C00-D48)**					
**Soft drinks, artificially sweetened**					
Deaths, n	4442	264	471	219	
Multivariable model, HR (95% CI)	1 (ref)	0.92 (0.81-1.05)	1.05 (0.95-1.16)	1.06 (0.92-1.22)	0.32
**Soft drinks, sugar sweetened**					
Deaths, n	4765	861	1164	348	
Multivariable model, HR (95% CI)	1 (ref)	0.97 (0.90-1.04)	0.99 (0.92-1.06)	1.01 (0.90-1.13)	0.87
**Circulatory diseases (ICD-10 codes I00-I99)**					
**Soft drinks, artificially sweetened**					
Deaths, n	1832	101	183	128	
Multivariable model, HR (95% CI)	1 (ref)	0.84 (0.69-1.04)	1.08 (0.92-1.27)	1.63 (1.35-1.96)	<.0001
**Soft drinks, sugar sweetened**					
Deaths, n	1983	513	614	215	
Multivariable model, HR (95% CI)	1 (ref)	1.00 (0.90-1.11)	0.96 (0.87-1.06)	1.06 (0.91-1.22)	0.59
**Digestive diseases (ICD-10 codes K00-K93)**					
**Soft drinks, artificially sweetened**					
Deaths, n	282	25	38	14	
Multivariable model, HR (95% CI)	1 (ref)	1.26 (0.82-1.93)	1.54 (1.07-2.20)	1.08 (0.62-1.88)	0.62
**Soft drinks, sugar sweetened**					

© 2019 Mullee A et al. *JAMA Internal Medicine.*

Deaths, n	302	60	73	42	
Multivariable model, HR (95% CI)	1 (ref)	0.94 (0.70-1.26)	1.07 (0.82-1.41)	1.49 (1.06-2.12)	0.02

HR = hazard ratio; CI = confidence interval. ICD-10 = International Classification of Diseases, 10th Revision. Multivariable model-Cox regression using body mass index (<22; 22-<25; 25-<30; 30-<35; ≥35 kg/m^2), physical activity index (inactive; moderately inactive; moderately active; active), education status (none; primary school completed; technical/professional school; secondary school; longer education including university; or not specified), alcohol consumption (non-consumers; <5; 5-<15; 15-<30; ≥30 g/day), smoking status and intensity (never; current , 1-15 cigarettes per day; current, 16-25 cigarettes per day; current, ≥16 cigarettes per day; former, quit ≤10 years; former, quit 11-20 years; former, quit ≥20 years; current, pipe/cigar/occasional; current/former, missing; unknown), smoking duration (<10; 10-<20; 20-<30; 30-<40; ≥40 years; smoking duration unknown), ever use of contraceptive pill (yes; no; or unknown), menopausal status (premenopausal; postmenopausal; perimenopausal/unknown menopausal status; or surgical postmenopausal), ever use of menopausal hormone therapy (yes; no; or unknown), and intakes of total energy (kcal/day), red and processed meat (g/day), and fruits and vegetables (g/day), coffee (g/day), juice (g/day) (all continuous), and stratified by age (1-year categories), center, and sex. Additionally, sugar sweetened and artificially sweetened soft drinks were mutually adjusted. Italy, Spain and Sweden are excluded from the sugar-sweetened and artificially-sweetened soft drinks analyses as information on type of soft drink consumption was not collected.

© 2019 Mullee A et al. *JAMA Internal Medicine.*

eTable 8. Multivariable Associations of Categories of Soft Drinks Consumption and All-Cause Mortality by Ascertainment of Death Method

	<1 glass/month	1–4 glasses/month	>1–6 glasses/week	1-<2 glasses/day	≥2 glasses/day	*ptrend*
**All-cause mortality**						
Linkage follow-up (Denmark, Italy, the Netherlands, Norway, Spain, Sweden, and UK)						
Total soft drinks, HR (95% CI)	1 (ref)	0.95 (0.92-0.98)	0.97 (0.94-1.00)	1.08 (1.02-1.13)	1.15 (1.09-1.21)	<.0001
Soft drinks, artificially-sweetened, HR (95% CI)	1 (ref)	0.94 (0.90-0.98)	1.00 (0.96-1.05)	0.92 (0.75-1.12)	1.26 (1.17-1.37)	<.0001
Soft drinks, sugar-sweetened, HR (95% CI)	1 (ref)	0.91 (0.88-0.95)	0.94 (0.91-0.98)	1.01 (0.94-1.10)	1.05 (0.97-1.13)	0.51
Active follow-up (France, Germany, Greece)						
Total soft drinks, HR (95% CI)	1 (ref)	1.04 (0.97-1.12)	1.08 (1.01-1.15)	1.32 (1.16-1.50)	1.28 (1.07-1.53)	<.0001
Soft drinks, artificially-sweetened, HR (95% CI)	1 (ref)	0.90 (0.76-1.07)	1.15 (1.00-1.32)	1.20 (0.91-1.58)	1.22 (0.90-1.67)	0.03
Soft drinks, sugar-sweetened, HR (95% CI)	1 (ref)	1.07 (0.99-1.15)	1.07 (0.99-1.15)	1.39 (1.20-1.63)	1.37 (1.10-1.71)	<.0001

HR = hazard ratio; CI = confidence interval. ICD-10 = International Classification of Diseases, 10th Revision. Multivariable model-Cox regression using body mass index (<22; 22-<25; 25-<30; 30-<35; ≥35 kg/m^2), physical activity index (inactive; moderately inactive; moderately active; active), education status (none; primary school completed; technical/professional school; secondary school; longer education including university; or not specified), alcohol consumption (non-consumers; <5; 5-<15; 15-<30; ≥30 g/day), smoking status and intensity (never; current , 1-15 cigarettes per day; current, 16-25 cigarettes per day; current, ≥16 cigarettes per day; former, quit ≤10 years; former, quit 11-20 years; former, quit ≥20 years; current, pipe/cigar/occasional; current/former, missing; unknown), smoking duration (<10; 10-<20; 20-<30; 30-<40; ≥40 years; smoking duration unknown), ever use of contraceptive pill (yes; no; or unknown), menopausal status (premenopausal; postmenopausal; perimenopausal/unknown menopausal status; or surgical postmenopausal), ever use of menopausal hormone therapy (yes; no; or unknown), and intakes of total energy (kcal/day), red and processed meat (g/day), fruits and vegetables (g/day), coffee (g/day), and fruit and vegetable juice (g/day) (all continuous), and stratified by age (1-year categories), center, and sex. Additionally, sugar sweetened and artificially sweetened soft drinks were mutually adjusted. Italy, Spain and Sweden are excluded from the sugar-sweetened and artificially-sweetend soft drinks analyses as information on type of soft drink consumption was not collected.

© 2019 Mullee A et al. *JAMA Internal Medicine.*

eTable 9. Multivariable Associations of Categories of Soft Drinks Consumption and Deaths Due to External Causes (ICD-10 Codes S00-Y98)

	**<1 glass/month**	**1–4 glasses/month**	**>1–6 glasses/week**	**≥1 glass/day**	***ptrend***
**External causes (ICD-10 codes S00-Y98)**					
**Overall soft drinks**					
Deaths, n	796	203	414	158	
Multivariable model, HR (95% CI)	1 (ref)	0.90 (0.76-1.05)	0.96 (0.84-1.09)	1.00 (0.83-1.20)	0.80
**Soft drinks, artificially sweetened**					
Deaths, n	807	91	85	26	
Multivariable model, HR (95% CI)	1 (ref)	0.86 (0.68-1.08)	0.93 (0.74-1.18)	1.05 (0.70-1.56)	0.85
**Soft drinks, sugar sweetened**					
Deaths, n	610	145	188	66	
Multivariable model, HR (95% CI)	1 (ref)	0.91 (0.75-1.10)	0.95 (0.79-1.14)	0.99 (0.75-1.31)	0.97

HR = hazard ratio; CI = confidence interval. ICD-10 = International Classification of Diseases, 10th Revision. Basic model-Cox regression with adjustment for total energy intake (kcal/day) and stratified by age (1-year categories), center, and sex. Multivariable model -Cox regression using body mass index (<22; 22-<25; 25-<30; 30-<35; ≥35 kg/m^2), physical activity index (inactive; moderately inactive; moderately active; active), education status (none; primary school completed; technical/professional school; secondary school; longer education including university; or not specified), alcohol consumption (non-consumers; <5; 5-<15; 15-<30; ≥30 g/day), smoking status and intensity (never; current , 1-15 cigarettes per day; current, 16-25 cigarettes per day; current, ≥16 cigarettes per day; former, quit ≤10 years; former, quit 11-20 years; former, quit ≥20 years; current, pipe/cigar/occasional; current/former, missing; unknown), smoking duration (<10; 10-<20; 20-<30; 30-<40; ≥40 years; smoking duration unknown), ever use of contraceptive pill (yes; no; or unknown), menopausal status (premenopausal; postmenopausal; perimenopausal/unknown menopausal status; or surgical postmenopausal), ever use of menopausal hormone therapy (yes; no; or unknown), and intakes of total energy (kcal/day), red and processed meat (g/day), fruits and vegetables (g/day), coffee (g/day), and fruit and vegetable juice (g/day) (all continuous), and stratified by age (1-year categories), center, and sex. Additionally, sugar sweetened and artificially sweetened soft drinks were mutually adjusted. Italy, Spain and Sweden are excluded from the sugar-sweetened and artificially-sweetened soft drinks analyses as information on type of soft drink consumption was not collected.

© 2019 Mullee A et al. *JAMA Internal Medicine.*
